# Defining production environments in U.S. sheep populations: clustering climate conditions and management practices

**DOI:** 10.1093/jas/skag239

**Published:** 2026-08-05

**Authors:** Hilal Yazar Gunes, Réka Howard, Thomas W Murphy, Carrie S Wilson, Bradley A Freking, Joan M Burke, Joshua Bret Taylor, Luiz F Brito, Ronald M Lewis

**Affiliations:** Department of Animal Sciences, University of Nebraska-Lincoln, Lincoln, NE 68583, United States; Department of Statistics, University of Nebraska-Lincoln, Lincoln, NE 68583, United States; Roman L. Hruska U.S. Meat Animal Research Center, USDA-ARS, Clay Center, NE 68933, United States; Range Sheep Production Efficiency Research Unit, USDA-ARS, Dubois, ID 83423, United States; Roman L. Hruska U.S. Meat Animal Research Center, USDA-ARS, Clay Center, NE 68933, United States; Dale Bumpers Small Farms Research Center, USDA-ARS, Booneville, AR 72927, United States; Range Sheep Production Efficiency Research Unit, USDA-ARS, Dubois, ID 83423, United States; Department of Animal Sciences, Purdue University, West Lafayette, IN 47907, United States; Department of Animal Sciences, University of Nebraska-Lincoln, Lincoln, NE 68583, United States

**Keywords:** climate, eco-management clusters, management survey, production environments, sheep

## Abstract

Sheep production in the United States spans diverse geographies and management practices, which are traditionally classified by climate alone. By combining information on climatic conditions and their management practices, we assigned groups of flocks to distinct eco-management clusters that characterized their shared production environments. Ninety-seven producers, whose geographically distributed flocks represented five breeds enrolled in the National Sheep Improvement Program, completed an online management survey. Breeds included Katahdin (*n* = 50), Polypay (*n* = 21), Suffolk (*n* = 13), Rambouillet (*n* = 8), and Targhee (*n* = 5). The questionnaire considered parasite control, lambing and udder health management, culling strategies, feeding methods, and climate mitigation practices. We characterized the climates at producer locations based on elevation, seasonal precipitation, soil moisture, and the Comprehensive Climate Index (CCI). The CCI integrated ambient temperature, humidity, wind speed, and solar radiation to reflect thermal stress. Clusters were first formed based on climate or management data via Principal Component Analysis and Multiple Correspondence Analysis, respectively. Both sources of information were then combined to form eco-management clusters using Factorial Analysis of Mixed Data. Cluster quality was assessed using silhouette widths. Differences among clustering approaches were tested with chi-square cross-tabulations and permutation tests. Flock assignments to climate and to management clusters were, statistically, independent within breeds (*P *> 0.16). Flock allocations to eco-management and management clusters, however, typically aligned (*P *≤ 0.01), although less so in Targhee–Rambouillet (*P *= 0.18). Such was also the case for climate-based clusters (*P *≤ 0.04) except in Suffolk (*P *= 0.50). Overall, coupling of climate and management appeared weakest in Suffolk flocks. Across eco-management clusters, differences in management practices were found for 26 husbandry practices in Katahdin, 21 in Polypay, 7 in Suffolk, and 5 jointly in Rambouillet and Targhee (*P *< 0.05). Although climate influenced husbandry strategies, producers often adopted unique management practices in response to local conditions. Collectively, climate and management defined distinct yet complementary drivers of environmental heterogeneity, supporting the concept of eco-management clustering as a holistic approach to characterizing the production environments of sheep flocks.

## Teaser

Production environments were more precisely defined by combining climatic conditions and producer-reported management practices into eco-management clusters for five major U.S. sheep breeds. Doing so provided a framework for re-evaluating the effects of environmental conditions on performance and understanding the impact of genotype-by-environment interactions in genetic evaluation schemes.

## Introduction

Sheep in the U.S. are raised across a wide spectrum of climates, from arid and semi-arid regions to temperate and subtropical areas. Substantial differences in flock management practices accompany this environmental variation. The nuances of these production systems are shaped by the interplay of genetic, environmental, management, and socioeconomic factors, creating both opportunities and challenges for improving the sustainability and productivity of U.S. sheep flocks. A key opportunity lies in this diversity itself. By understanding how climate and management interact to shape animal performance, producers can actively manage these environments to improve the health and productivity of their flocks.

At the same time, environmental variability can present challenges when interactions between an animal’s genotype and its production environment—known as genotype-by-environment interactions (G × E)—occur. Such G × E have been detected in lambs for parasite resistance and body weight at weaning (Arisman et al. 2023), for average daily gain and feed efficiency (Lewis et al. 2006), and for ultrasonic measures of muscle and fat depth near harvest (Márquez et al. 2015). Still, accurately describing production environments contributing to G × E is difficult, as traditional classification methods often fail to capture the full scope of environmental influences.

Traditional classifications of production environments have relied primarily on broad regional groupings or climate-based delineations (McManus et al. 2021; Rowan et al. 2021). McManus et al. (2021) also suggested that regional climatic delineations are often consistent across species. However, this assumption may not always hold since environmental classifications based solely on climate overlook the role of management. Even within flocks that have similar climatic conditions, differences in feeding regimes, shelter, parasite control measures, and other husbandry practices can create distinct production environments. Thus, for a more comprehensive classification of production environments, a framework that integrates both climate and management factors may be necessary.

Recent work in other livestock species supports the value of integrating climate and management information when defining production environments. Santana et al. (2025) demonstrated in pasture-raised beef cattle that farm-level environmental and management descriptors, including climate classification, soil type, elevation, and producer-reported management practices, accounted for a considerable share of between-farm variation in performance traits. Their findings highlight that biologically informed environmental descriptors can improve the characterization of production environments and the modeling of G × E. However, such approaches have rarely been applied in sheep populations or extended to define discrete production environments through clustering methods that explicitly integrate climate and management information.

We hypothesized that by systematically combining information on climate and management practices we more effectively capture differences among production environments than with climate or management alone. In testing this hypothesis, we considered four main U.S. sheep breed-types: fine-wool, semi-prolific maternal, terminal sire, and hair. We evaluated climate and management data collected on flocks enrolled in the National Sheep Improvement Program (NSIP), which were spread geographically across the U.S. Our main objectives were to: 1) establish a framework for grouping or clustering flocks within each breed-type based on similarities in their climates (eco clusters), management practices (management clusters), and the combined effects of both (eco-management clusters) and 2) compare the efficacies of these clusters in capturing differences in production environments.

## Materials and methods

### Animal care and use

This study involved preparation and distribution of an online survey to NSIP producers. The survey was reviewed and approved by the University of Nebraska-Lincoln Institutional Review Board (IRB), with survey responses stored in accordance with IRB approval and standards (IRB Protocol Number: 20231123225EX).

### Data collection

Climate and management data were collected on 97 NSIP flocks representing five major sheep breeds: Katahdin (50 flocks), Polypay (21 flocks), Suffolk (13 flocks), Rambouillet (8 flocks), and Targhee (5 flocks). The flocks surveyed represented the four main breed-types in the U.S. sheep industry: hair (Katahdin), semi-prolific maternal (Polypay), terminal sire (Suffolk), and fine-wool (Rambouillet and Targhee). Rambouillet and Targhee flocks were considered together in our analysis, given their genetic and management similarities, and to increase the sample size for fine-wool breeds. Further description of these breed-types and breeds is provided in Yazar Gunes et al. (2026).

#### Climate data

Climate data were sourced from NASA Prediction of Worldwide Energy Resource (NASA POWER 2024) based on each flock’s latitude and longitude coordinates. Flock level data were elevation (m above sea level), average monthly total precipitation (mm), which included both rainfall and snowfall with snowfall converted to its equivalent depth of water, and average monthly soil moisture (cm³ water/cm³ soil volume). Additionally, meteorological variables required to calculate CCI, specifically ambient temperature (°C), relative humidity (%), wind speed (m/s), and solar radiation (W/m²), were obtained at 2 m above ground level, which is the minimum above ground measurement height available in NASA POWER. Although this height exceeds the stature of sheep, the CCI was recorded in a consistent fashion across all flock locations and thereby provided a comparative indicator of thermal environments experienced by animals. Climate data from a recent 5-yr period (2018–2022) were obtained, as our intent was to characterize the conditions experienced by each flock concurrent with the flock’s owner reporting of their management practices.

#### Management data

Management data were collected from a survey designed with input from NSIP members selected to represent the perspectives of their breed-type(s). Recruitment for participation in the survey involved two steps. In November 2023, an email was sent to 242 NSIP producers raising a targeted breed to both describe the survey and invite them to participate. Producers who expressed interest were sent another email with a link to the survey that we developed in Qualtrics (Qualtrics 2023), a secure web-based platform commonly used for survey research. The survey remained open through February 2024. Overall, 97 producers completed the survey (40.1% response rate). Breed-specific response rates were 47.2% (Katahdin), 36.8% (Polypay), 44.8% (Suffolk), 42.1% (Rambouillet), and 16.1% (Targhee).

The survey consisted of 60 questions (Supplementary Material), with the first three collecting personal information (eg flock number, address). The remaining 57 questions were designed to address specific management practices. The questions were divided into seven sections: general flock management (11 questions), parasite control (17 questions), lambing management (7 questions), udder health management (6 questions), culling strategies (2 questions), feeding methods (8 questions), and climate mitigation strategies (eg availability of housing and other facilities; 6 questions). Four questions—Q21 and Q22 (parasite management), Q32 (lambing management), and Q43 (udder health management)—had insufficient responses and were excluded, leaving 53 for analyses. Further information about the construction of the survey and the responses received is detailed in Yazar Gunes et al. (2026).

### Data processing

#### Climate data processing

Climate variables were processed to compute CCI values following the methodology described by Mader et al. (2010). Monthly values of precipitation, soil moisture, and CCI were first aggregated to seasonal averages for each flock, and these seasonal values were then averaged over the 5-yr period (2018–2022). Seasons were defined as winter (December 1–February 28/29), spring (March 1–May 31), summer (June 1–August 31), and fall (September 1–November 30), following standard meteorological conventions (3 full calendar months per season). Additionally, to ensure comparability in multivariate analyses, all climate variables were standardized (mean-centered and scaled to unit variance).

To facilitate cluster interpretation, elevation, seasonal CCI, precipitation, and soil moisture values were grouped into descriptive categories. Elevation was classified into low (<500 m), moderate (≥500–≤1,000 m), and high (>1,000 m) categories. Median elevation was reported in cluster summaries because elevation was right-skewed, and the median provided a more representative measure of the central tendency than the mean. Seasonal precipitation, soil moisture, and CCI were separated into three categories (low, moderate, and high) by dividing the distribution of each variable within each season across all breeds and flocks into terciles (lower third, middle third, and upper third; Table 1). This approach ensured that climatic categories were directly comparable across breeds and regions. These categorical variables were used solely for descriptive summaries of the clusters. All statistical clustering procedures were conducted using standardized climate variables measured on a continuous scale.

**Table 1:** Global tercile thresholds for seasonal climate variables across all flocks.^1^

Season	Variable	Low	Moderate	High
**Fall**	CCI, °C	−3.73, 7.12	7.13, 10.82	11.03, 27.02
	Precipitation, mm	12.57, 62.92	63.86, 80.24	80.42, 188.63
	Soil moisture, cm³/cm³	0.28, 0.52	0.53, 0.60	0.61, 0.73
**Spring**	CCI, °C	−4.54, 5.08	5.09, 8.42	8.55, 25.25
	Precipitation, mm	7.39, 72.56	72.57, 90.79	91.13, 156.40
	Soil moisture, cm³/cm³	0.28, 0.63	0.64, 0.79	0.80, 0.89
**Summer**	CCI, °C	9.96, 22.42	22.49, 25.43	25.88, 34.78
	Precipitation, mm	19.14, 86.23	86.24, 98.35	98.87, 175.63
	Soil moisture, cm³/cm³	0.21, 0.51	0.52, 0.61	0.62, 0.72
**Winter**	CCI, °C	−20.42, −9.39	−9.29, −5.65	−5.50, 17.72
	Precipitation, mm	6.22, 31.15	31.16, 74.36	74.37, 274.57
	Soil moisture, cm³/cm³	0.38, 0.58	0.59, 0.76	0.77, 0.90

1 The minimum and maximum values of the thresholds reported.

Cluster summaries were then generated using the most common category for a climatic variable among flocks assigned to each cluster. The numbers of flocks within a cluster with that most common category were determined. If, for instance, three of five flocks within a cluster were categorized as high, it was reported as “High (3/5).”

#### Management data processing

Since survey data primarily consisted of categorical responses, they were transformed into factor variables for statistical analyses. For “check-all-that-apply” questions, responses were harmonized by collapsing multiple selections into a small set of mutually exclusive, biologically coherent categories. This was done to increase the number of responses (counts) within categories and to improve the stability and interpretability of the analyses.

For variables reported on a whole-flock basis (eg total anthelmintic use, mastitis treatment costs), larger flocks would naturally be expected to report higher values. Flock sizes were reported in categorical ranges (< 16, 16–30, 31–50, 51–100, 101–250, and > 250 ewes) rather than as exact numbers of ewes since those counts may vary over time. Similarly, land area was recorded as categorical ranges (<4, 4–20, 21–40, 41–200, 201–405, and >405 ha) rather than exact acreage. Consequently, these variables could not be standardized to a per-ewe basis. Because flock sizes and land areas were not collected as exact values, overall averages or exact ranges could not be reported. Survey responses reported on a whole-flock basis, therefore, must be interpreted cautiously to avoid attributing the differences observed as anything more than differences in flock sizes and corresponding acreages.

### Multivariate analysis and clustering

Multivariate clustering techniques were used to delineate production environments based on climate and management characteristics alone or in combination. These analyses were conducted in the R software (R Core Team 2023) using several packages. Principal Component Analysis (PCA), Multiple Correspondence Analysis (MCA), and Factorial Analysis of Mixed Data (FAMD) were conducted with “FactoMineR” (Lê et al. 2008). Using this same R-package, clusters were delineated using Hierarchical Clustering on Principal Components (HCPC) using the retained component scores from PCA, MCA, or FAMD analyses. For each clustering approach, the optimal number of clusters was determined using the default criterion in the FactoMineR procedure, implemented through the HCPC algorithm, with the partitions based on changes in the percentage of variation captured between-clusters. Additional R packages used included factoextra (Kassambara and Mundt 2020) for visualization and interpretation of multivariate and clustering results, cluster (Maechler et al. 2023) for silhouette width computation and cluster evaluation, MASS (Venables and Ripley 2002) for Linear Discriminant Analysis (LDA), and ggplot2 (Wickham 2016) and maps (Becker et al. 2023) for geographic visualization of flock locations and cluster assignments. Three clustering approaches were implemented: 1) eco clustering, which classified production systems based on climate data alone, 2) management clustering, which used only management data, and 3) eco-management clustering, which combined climate and management data.

The flocks participating in the survey were distributed across the U.S., as shown in Figure 1. However, individual breed-types were concentrated in different regions. Polypay and Suffolk flocks were in the Upper Midwest and Northeast, while Rambouillet and Targhee producers were mainly in the Intermountain West and Great Plains. Katahdin producers were most widely scattered geographically but with greatest concentrations in the Midwest, Northeast, and Southeast regions. Given this regional distribution, unsurprisingly, management practices differed across breed-types (Yazar Gunes et al. 2026). However, we also anticipated that husbandry strategies would differ amongst flocks within each breed-type both within and across regions. The multivariate analyses, therefore, were conducted separately within each breed-type.

**Figure 1:**
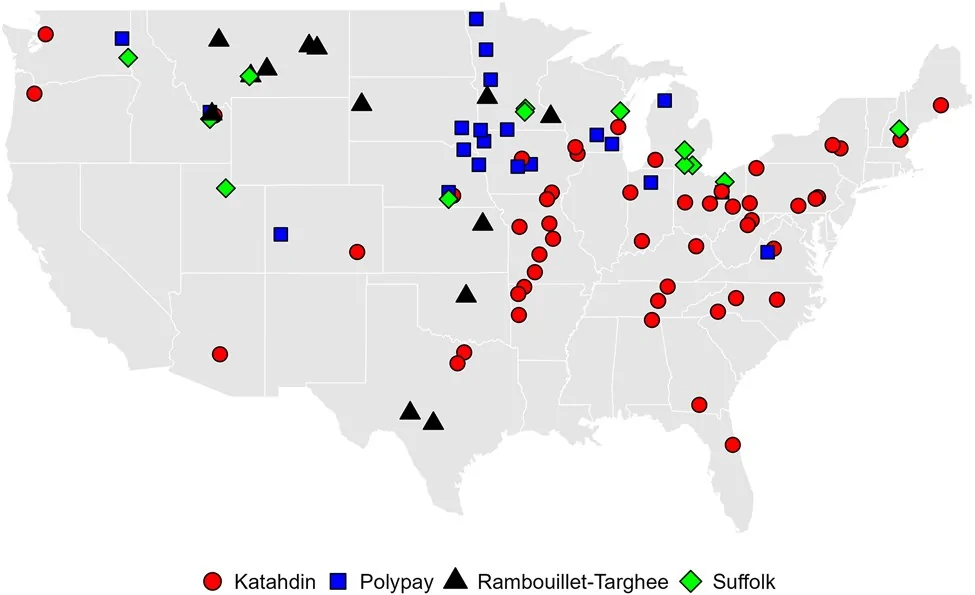
Distribution of the flocks completing the survey across breeds-types.

#### Climate-based clustering (eco clusters)

Eco clustering focused solely on local environmental factors to identify distinct climate-driven production systems. Only climatic data on flocks that completed the survey were considered. As described in Aluja et al. (2018), we first applied PCA to reduce the dimensionality of the climate dataset, retaining principal components until they collectively explained at least 70% of the total variance. These principal components were then used in HCPC to classify flocks into eco clusters.

#### Management-based clustering (management clusters)

Management practices gleaned from the survey responses were used to form management clusters using MCA. This approach is well-suited for identifying patterns in categorical data (Atkinson 2024). To interpret the results, we examined the variables with the highest contributions (v.test values) to each MCA dimension (the underlying axes produced by the MCA) and cluster. Specifically, variables were considered influential if their absolute v.test value exceeded 2.0, corresponding approximately to a 5% significance threshold. Variables with stronger contributions were treated as primary drivers of cluster separation, whereas variables with smaller or non-significant v.test values were not considered central to cluster characterization. This approach ensured that cluster descriptions were based on the most influential management practices, while avoiding over-interpretation of marginal factors.

We also cross-checked cluster assignments with the underlying survey data to confirm that they distinguished common practices among flocks. Because MCA characterizes associations among categorical response levels rather than the contribution of individual variables to the variation, our interpretations of management cluster emphasized integrated patterns across multiple sections of the survey. Consequently, in contrast to eco and eco-management clusterings, which allowed clusters to be characterized through tabular summaries of dominant climatic or combined variables, management clusters were summarized narratively.

#### Combined climate- and management-based clustering (eco-management clusters)

Lastly, to develop a more comprehensive classification of production environments, we conducted FAMD (Lê et al. 2008) to integrate both the climate and management information. This approach accommodates datasets that combine continuous (climate) and categorical (management) variables and was well suited for capturing interactions between climatic conditions and producer-driven husbandry practices. The resulting flock scores, which represent projections onto principal components, were used as inputs for HCPC to define eco-management clusters.

Following FAMD-based clustering, LDA was applied to evaluate the discriminative power of variables shaping the eco-management clusters. We focused on those variables with the largest standardized coefficients on the first two discriminant functions, which captured the strongest contrasts among clusters. To enhance interpretability, only the top 5% of contributing variables were retained for characterization. This reduction step was applied only to FAMD-derived clusters, as the number of climate variables was limited and the management clustering via MCA already reduced the dimensionality of categorical variables.

While FAMD, followed by HCPC, provided the statistical grouping of flocks, it did not reveal which climatic conditions or management practices defined each cluster. The loadings indicated which variables contributed to the formation of the axes, but they did not show how those variables were manifested within clusters. Therefore, we used LDA to rank variables by their discriminative power and then summarized cluster-level responses to translate clusters into biologically meaningful descriptions. This ensured that eco-management classifications were shaped by the most relevant climatic and management factors influencing sheep production systems in the United States.

For interpretation, we summarized management responses and climate conditions within each cluster by calculating the proportion of flocks associated with each categorical response and the standardized means for continuous variables. These summaries were then used to characterize management practices and climatic conditions for each eco-management cluster.

#### Cluster assignment validation

To assess cluster quality, we compared average silhouette width (Rousseeuw 1987) across PCA-, MCA-, and FAMD-derived clusters. To evaluate interpretability, Fisher’s exact tests were used to determine whether the 53 management variables significantly differed across eco-management clusters. These tests were used only for the eco-management clustering approach because management variables did not contribute to our evaluation of eco clusters. Rather than serving as a measure of clustering quality, the number of significant variables was used descriptively to summarize the breadth of management differentiation across clusters. It was not intended to imply that a larger number of significant variables meant better assignments of flocks to clusters.

## Results

### Clustering

#### Assignments

Flocks were assigned to clusters based on their climate (using PCA), management practices (using MCA), or the combination of both (using FAMD). Although there were considerable differences in the number of flocks within the breed-types considered in the study, the number of clusters formed both within and among sources of information about production environments was similar (Table 2).

**Table 2:** Number of clusters per clustering method, and number of flocks contributing climate and management information to form those clusters.

Breed	No. of eco clusters	No. of management clusters	No. of eco-management clusters	No. of flocks contributed to the formation of clusters
**Katahdin**	4	5	6	50
**Polypay**	3	6	6	21
**Rambouillet–Targhee**	5	6	6	13^1^
**Suffolk**	4	6	4	13

1 Of the 13 flocks, 8 were Rambouillet and 5 were Targhee.

#### Inertia

To summarize the multivariate structure of the climate, management, and combined datasets, we determined the percentage of variation (inertia) captured within each method used to reduce dimensionality. As PCA, MCA, and FAMD differ in how variance is defined and distributed, these inertia values were interpreted only within a method and not used for cross-method comparisons. For the climate-only PCA, the cumulative inertia of the first five principal components was high, ranging from 93.9% in Katahdin to 99.2% in Suffolk, reflecting the concentration of variance in a few of the climate principal component axes. In MCA and FAMD, inertia was distributed across many categorical indicator variables, resulting in lower inertia values as is typically found with these methods (MCA: 29.6% in Katahdin, 43.0% in Polypay, 55.0% in Rambouillet–Targhee, and 55.9% in Suffolk; FAMD: 30.5% in Katahdin, 43.1% in Polypay, 57.4% in Rambouillet–Targhee, and 56.8% in Suffolk).

#### Validation

Cluster structure was validated using average silhouette widths across PCA-, MCA-, and FAMD-derived clusters (Table 3). Polypay, Rambouillet–Targhee, and Suffolk flocks showed a similar pattern, with eco-based clustering demonstrating the strongest cohesion relative to management-only and eco-management approaches. In contrast, for Katahdin flocks, clustering based on management or eco-management outperformed eco clustering. Overall, silhouette widths generally exceeded the threshold indicating weak clustering structure (> 0.25). However, none reached the threshold considered as strong clustering (> 0.50). Those threshold values are commonly used as interpretive guidelines for mean silhouette coefficients (Kaufman and Rousseeuw 1990) and have been applied in other recent studies (eg Benaya et al. 2023).

**Table 3:** Average silhouette widths for eco-, management-, and eco-management-based clusters by sheep breed.

Breed	Eco based	Management based	Eco-management based
**Katahdin**	0.351	0.471	0.447
**Polypay**	0.331	0.303	0.272
**Rambouillet–Targhee**	0.455	0.239	0.288
**Suffolk**	0.450	0.173	0.164

To further assess the interpretability of eco-management clusters, we tested whether management practices differed across FAMD-derived groups. We used the Fisher’s exact test to characterize differences in producer responses for each of the 53 management survey questions associated with flock assignments to distinct eco-management clusters. Management practices differed (*P *< 0.05) across eco-management clusters for 26 questions in Katahdin, 21 questions in Polypay, 7 questions in Suffolk, and 5 questions in Rambouillet–Targhee. Among the management variables, differences across eco-management clusters were primarily driven by parasite management practices, particularly gastrointestinal nematode (GIN) parasite control programs, although cluster formation also reflected underlying climatic variation. In Katahdin and Polypay, nearly half of the distinguishing questions coincided with parasite control (46% and 52%, respectively), with the additional differences due to general and feeding management practices. In Suffolk, the variation in flock assignments were overwhelmingly associated with differences in parasite management practices (71%). In Rambouillet–Targhee, with the fewest significant differences, flocks were assigned to eco-management clusters based on a mix of their general, parasite, and feeding management practices. The dominance of the parasite control strategies amongst the management practices that distinguished eco-management clusters may in part have reflected its predominance in the survey (30% of the questions), although undoubtedly it also reflected sheep producers’ focus on GIN parasitism in their husbandry regimes. A list of the survey questions associated with the differences in flock assignments to eco-management clusters is provided in Table S1.

#### Consistency of flock assignments

The consistency of flock assignments to clusters based on climate, management, or their combination, was tested pairwise with chi-squared and permutation-based tests (Table 4). Based on the more conservative permutation tests, regardless of breed, the same subset of flocks was not consistently clustered together (independent) when comparing clusters based on climate or on management alone (*P *> 0.16). However, there was clear overlap (dependency) between cluster assignments based on climate only and on climate and management combined in Katahdin (*P *< 0.001), Polypay (*P *= 0.04), and Rambouillet–Targhee (*P *< 0.001), whereas no such association was detected in Suffolk (*P *= 0.50). Furthermore, a co-dependency of flock assignments to the same management and eco-management clusters was found in Katahdin (*P *< 0.001), Polypay (*P *< 0.001), and Suffolk (*P *= 0.01) but not in Targhee–Rambouillet (*P *= 0.18). These patterns are illustrated in Figures 2–5, and in Table 5. In Katahdin and Polypay, most flocks remained grouped together when eco-management clustering was compared with management-only clustering; there was greater reassignment when comparing eco with eco-management clusters. Conversely, in Rambouillet–Targhee flocks, assignments to the same eco-management and eco clusters were more consistent than to management clusters. The least consistent assignments across the three clustering approaches were in Suffolk. Overall, such breed-specific reassignment patterns indicated that eco-management clusters captured more breed-specific differences in flock groupings than climate or management alone.

**Figure 2:**
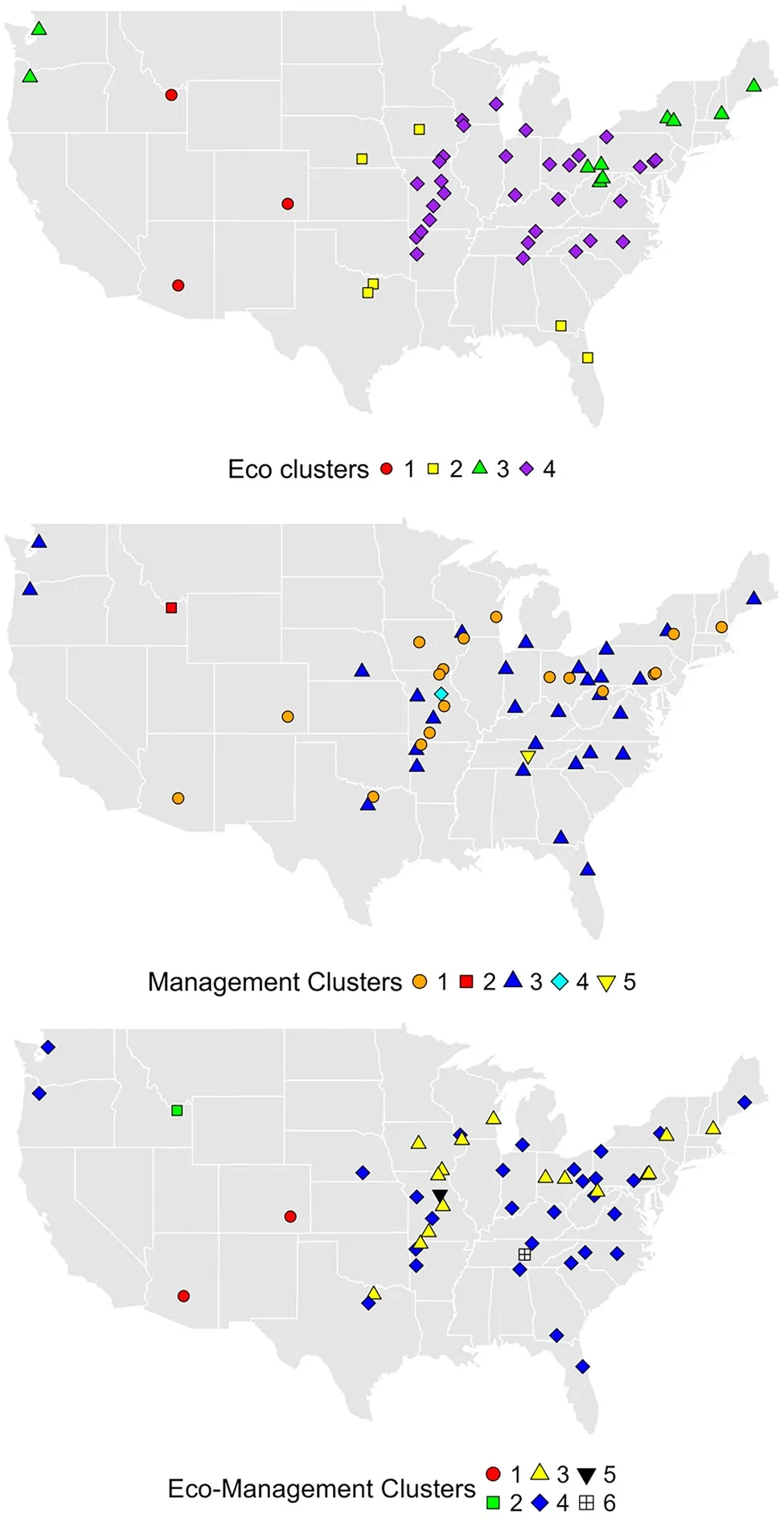
Geographic distribution of Katahdin flocks based on eco clusters (top), management clusters (middle), and eco-management clusters (bottom). Each dot represents a flock, with color and shape denoting its assigned cluster.

**Figure 3:**
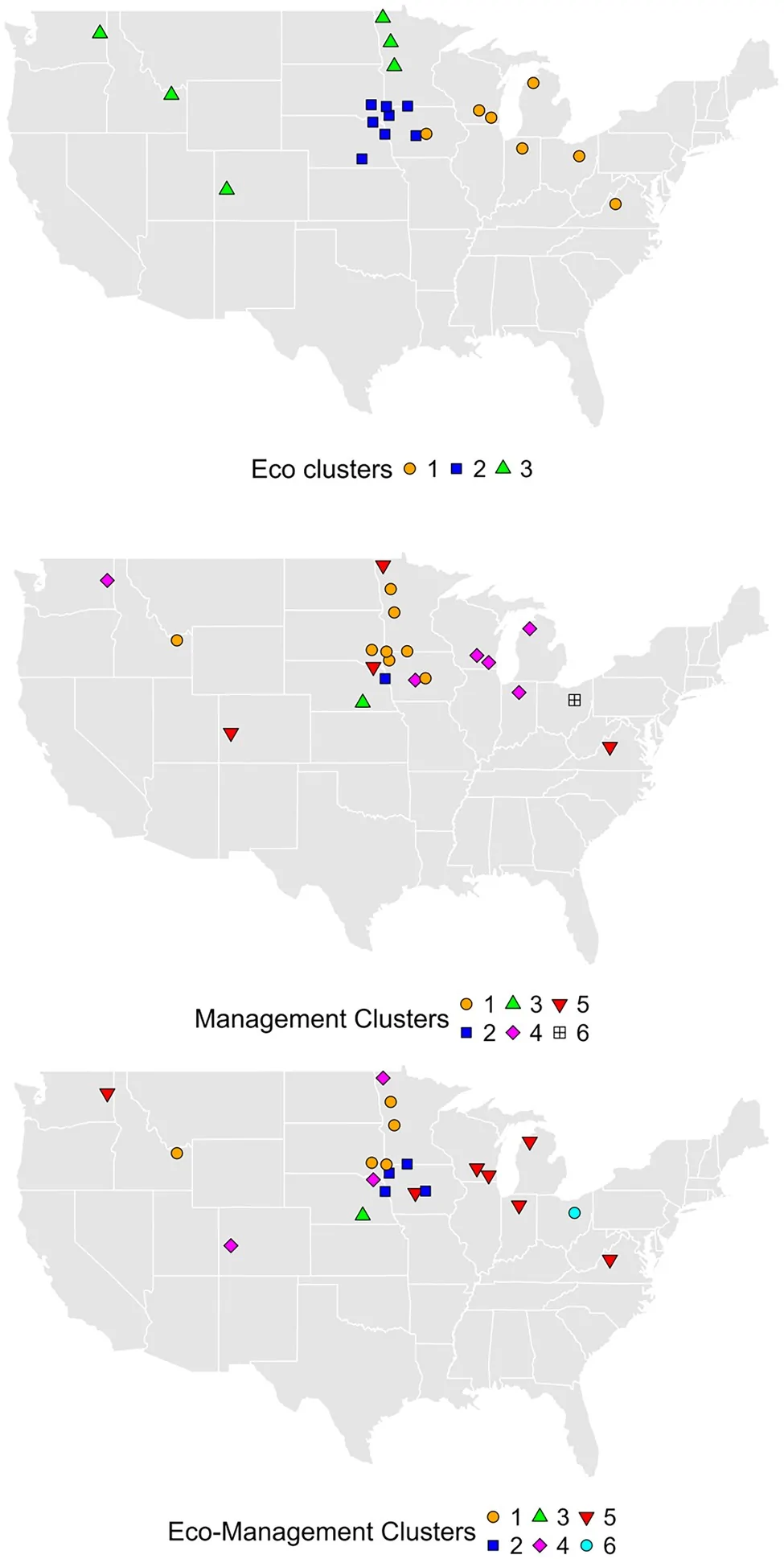
Geographic distribution of Polypay flocks based on eco clusters (top), management clusters (middle), and eco-management clusters (bottom). Each dot represents a flock, with color and shape denoting its assigned cluster.

**Figure 4:**
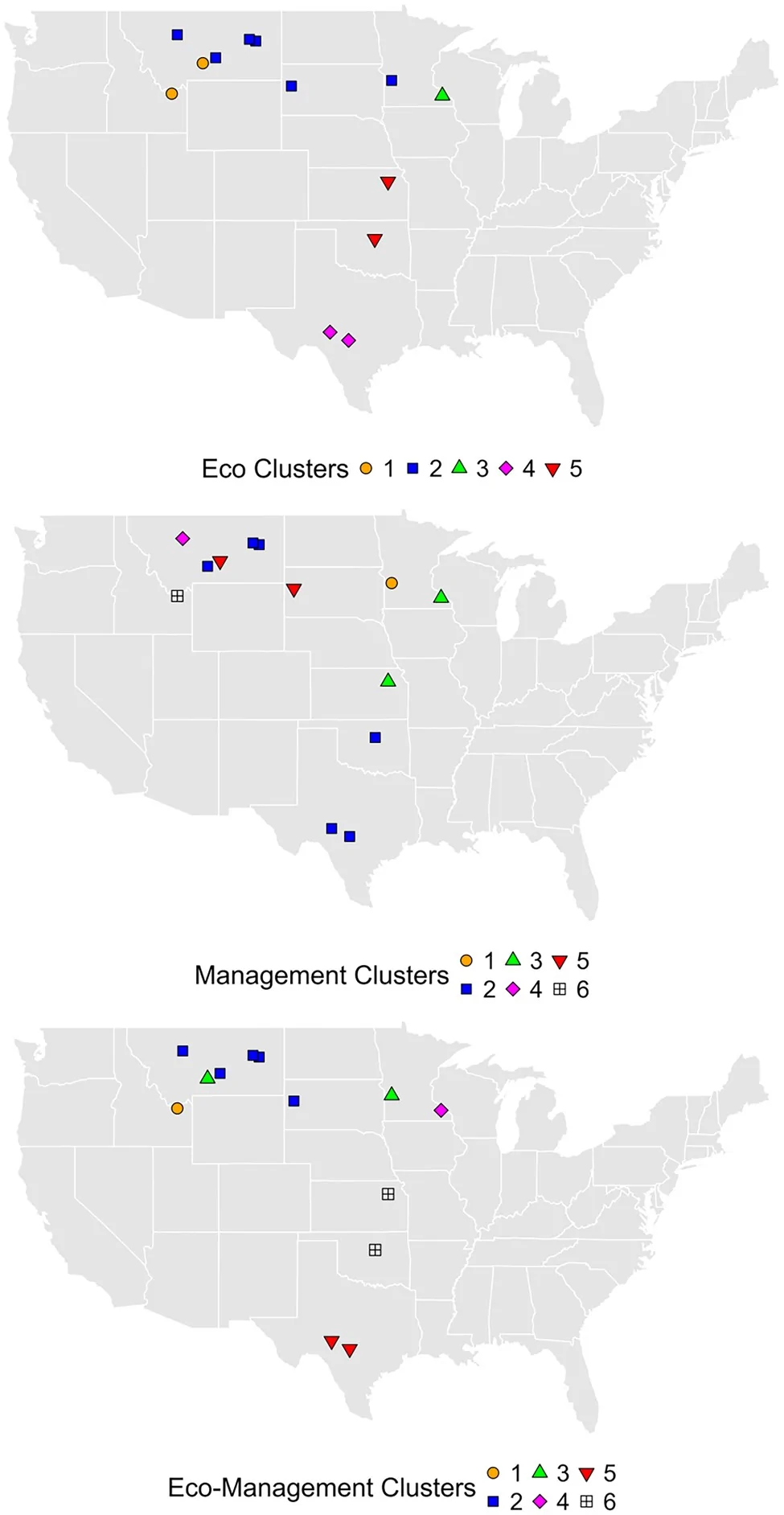
Geographic distribution of Rambouillet–Targhee flocks based on eco clusters (top), management clusters (middle), and eco-management clusters (bottom). Each dot represents a flock, with color and shape denoting its assigned cluster.

**Figure 5:**
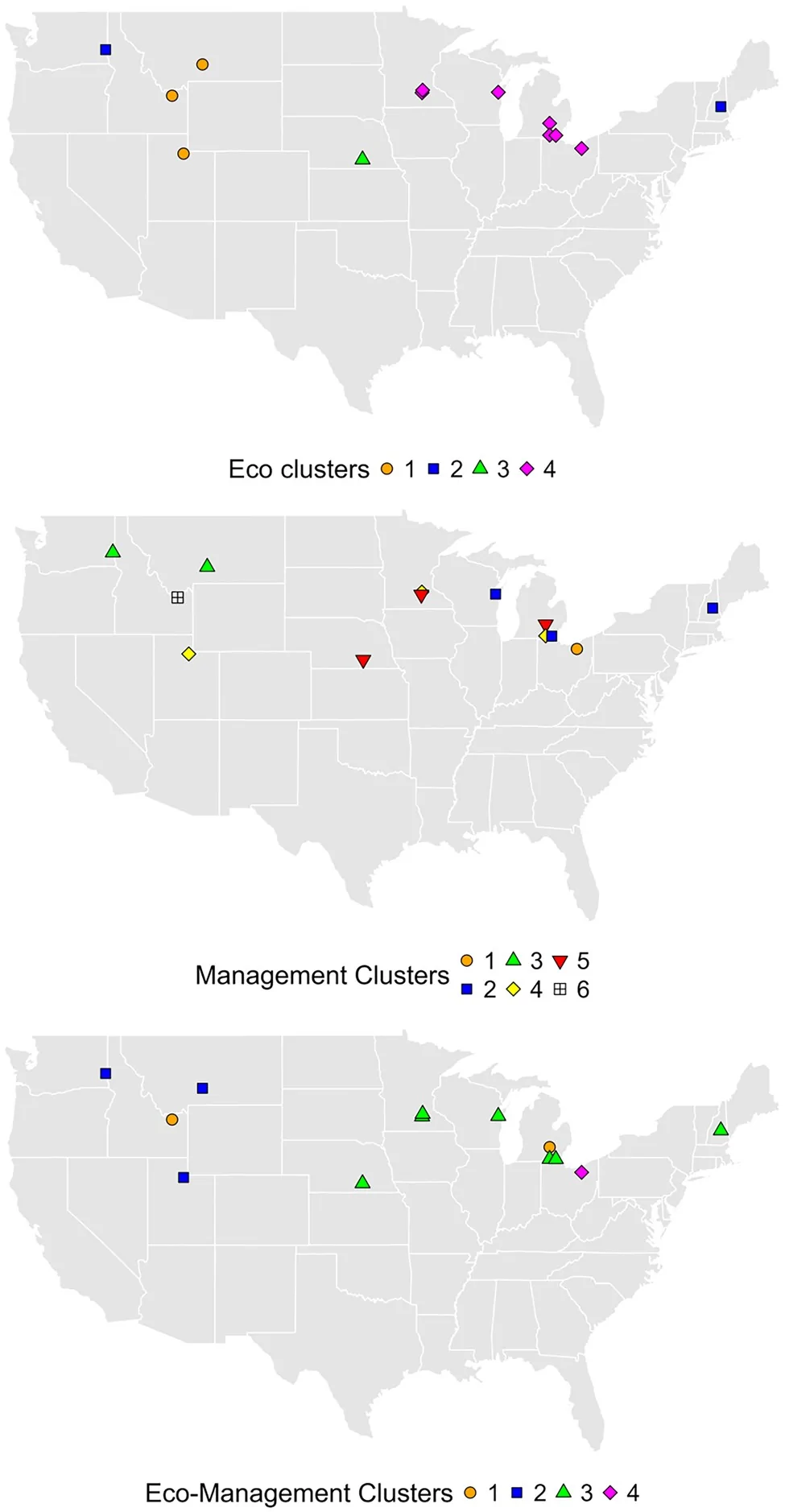
Geographic distribution of Suffolk flocks based on eco clusters (top), management clusters (middle), and eco-management clusters (bottom). Each dot represents a flock, with color and shape denoting its assigned cluster.

**Table 4:** Statistical comparisons of flock classifications across clustering methods using chi-squared and permutation tests for each sheep breed.

Breed and clusters compared	Degrees of freedom	Chi-square statistic	Chi-square *P*-value	Range chi-square values	Permutation *P*-value
**Katahdin**					
**Eco vs. management**	12	19.86	0.07	2.08, 44.08	0.16
**Eco vs. eco-management**	15	51.39	<0.001	3.34, 54.89	<0.001
**Management vs. eco-management**	20	200	<0.001	4.49, 150.13	<0.001
**Polypay**					
**Eco vs. management**	10	10.74	0.38	6.11, 26.30	0.40
**Eco vs. eco-management**	10	16.81	0.08	4.62, 28.63	0.04
**Management vs. eco-management**	25	79.78	<0.001	10.93, 60.92	<0.001
**Rambouillet–Targhee**					
**Eco vs. management**	20	19.86	0.47	11.19, 44.78	0.67
**Eco vs. eco-management**	20	47.67	<0.001	11.27, 45.07	<0.001
**Management vs. eco-management**	25	32.28	0.15	13.87, 50.92	0.18
**Suffolk**					
**Eco vs. management**	15	13.98	0.53	9.08, 39.00	0.63
**Eco vs. eco-management**	9	9.32	0.41	3.83, 29.57	0.50
**Management vs. eco-management**	15	29.30	0.02	9.08, 39.00	0.01

**Table 5:** Consistency in flock assignments to clusters across clustering approaches.^1^

Comparison (eco-management as baseline)	Same assignment, *n* (%)	Different assignments, *n* (%)
**Katahdin**		
**Eco-management vs. eco**	34 (68.0)	16 (32.0)
**Eco-management vs. management**	50 (100.0)	0 (0.0)
**Polypay**		
**Eco-management vs. eco**	15 (71.4)	6 (28.6)
**Eco-management vs. management**	19 (90.5)	2 (9.5)
**Rambouillet–Targhee**		
**Eco-management vs. eco**	12 (92.3)	1 (7.7)
**Eco-management vs. management**	8 (61.5)	5 (38.5)
**Suffolk**		
**Eco-management vs. eco**	9 (69.2)	4 (30.8)
**Eco-management vs. management**	7 (53.8)	6 (46.2)

1 Flocks were classified as “same” if assigned to the same cluster under both approaches, and “different” if assigned to different clusters.

### Climate-based clustering (eco clusters)

Climate-based clustering generated three to five distinct production environments within each breed (Figures 2–5, top panels), which were driven by variation in elevation and seasonal CCI, precipitation, and soil moisture. Across breeds, the first principal component primarily reflected gradients in seasonal precipitation and soil moisture, whereas the second component was largely associated with seasonal CCI and elevation. A summary of the climatic factors captured by these eco clusters is provided in Table 6. Eco clusters ranged from high-elevation systems with cold winters and either moderate or low precipitation (eg Rambouillet–Targhee eco cluster 1, Suffolk eco cluster 1) to low-elevation systems with more variable precipitation patterns and generally moderate to high seasonal CCI values (eg Katahdin eco clusters 3 and 4, Rambouillet–Targhee eco cluster 5).

**Table 6:** Summary of eco clusters defined within each breed, based on median elevation, and seasonal CCI values, precipitation, and soil moisture.

	Eco cluster					
Breed	Category	No. of flocks	Elevation^1^^,^^2^	Seasonal CCI values^2^^,^^3^	Seasonal precipitation^2^^,^^3^	Seasonal soil moisture^2^^,^^3^
**Katahdin**	1	3	High (2/3)	Wi: High (2/3); Sp: High (2/3); Su: High (2/3); Fa: High (2/3)	Wi: Low (3/3); Sp: Low (3/3); Su: Low (3/3); Fa: Low (3/3)	Wi: Low (3/3); Sp: Low (3/3); Su: Low (3/3); Fa: Low (3/3)
	2	6	Low (5/6)	Wi: High (4/6); Sp: High (5/6); Su: High (6/6); Fa: High (5/6)	Wi: Mod. (3/6); Sp: Mod. (3/6); Su: Low (2/6); Fa: Mod. (4/6)	Wi: Low (4/6); Sp: Low (4/6); Su: Low (5/6); Fa: Low (3/6)
	3	10	Low (9/10)	Wi: Mod. (7/10); Sp: Low (10/10); Su: Low (10/10); Fa: Low (9/10)	Wi: High (10/10); Sp: Mod. (5/10); Su: Low (5/10); Fa: High (7/10)	Wi: High (6/10); Sp: Mod. (5/10); Su: Mod. (6/10); Fa: Mod. (7/10)
	4	31	Low (31/31)	Wi: High (20/31); Sp: High (18/31); Su: Mod. (13/31); Fa: High (17/31)	Wi: High (17/31); Sp: High (23/31); Su: High (20/31); Fa: High (20/31)	Wi: High (21/31); Sp: High (20/31); Su: High (23/31); Fa: High (22/31)
**Polypay**	1	7	Low (6/7)	Wi: Low (3/7); Sp: Mod. (5/7); Su: Low (3/7); Fa: Mod. (6/7)	Wi: Mod. (4/7); Sp: Mod. (6/7); Su: Mod. (5/7); Fa: Mod. (4/7)	Wi: Mod. (7/7); Sp: Mod. (5/7); Su: Mod. (5/7); Fa: Mod. (4/7)
	2	8	Low (7/8)	Wi: Low (6/8); Sp: Mod. (7/8); Su: High (6/8); Fa: Mod. (7/8)	Wi: Low (8/8); Sp: Low (6/8); Su: Mod. (4/8); Fa: Low (6/8)	Wi: Low (7/8); Sp: Low (8/8); Su: Low (6/8); Fa: Low (7/8)
	3	6	Low (3/6)	Wi: Low (4/6); Sp: Low (5/6); Su: Low (3/6); Fa: Low (6/6)	Wi: Low (4/6); Sp: Low (6/6); Su: Low (6/6); Fa: Low (6/6)	Wi: Low (4/6); Sp: Low (5/6); Su: Low (4/6); Fa: Low (4/6)
**Rambouillet–Targhee**	1	2	High (2/2)	Wi: Low (2/2); Sp: Low (2/2); Su: Low (2/2); Fa: Low (2/2)	Wi: Low (1/2); Sp: Low (2/2); Su: Low (2/2); Fa: Low (2/2)	Wi: Low (2/2); Sp: Low (1/2); Su: Low (1/2); Fa: Low (1/2)
	2	6	Mod. (3/6)	Wi: Low (3/6); Sp: Mod. (4/6); Su: Mod. (4/6); Fa: Low (5/6)	Wi: Low (6/6); Sp: Low (6/6); Su: Low (5/6); Fa: Low (6/6)	Wi: Low (6/6); Sp: Low (6/6); Su: Low (6/6); Fa: Low (6/6)
	3	1	Low (1/1)	Wi: Low (1/1); Sp: Low (1/1); Su: Mod. (1/1); Fa: Low (1/1)	Wi: Low (1/1); Sp: Mod. (1/1); Su: High (1/1); Fa: Mod. (1/1)	Wi: Mod. (1/1); Sp: Mod. (1/1); Su: High (1/1); Fa: High (1/1)
	4	2	Mod. (2/2)	Wi: High (2/2); Sp: High (2/2); Su: High (2/2); Fa: High (2/2)	Wi: Low (2/2); Sp: Low (2/2); Su: Low (2/2); Fa: Low (2/2)	Wi: Low (1/2); Sp: Low (2/2); Su: Low (2/2); Fa: Mod. (2/2)
	5	2	Low (2/2)	Wi: High (2/2); Sp: High (2/2); Su: High (2/2); Fa: High (2/2)	Wi: Low (1/2); Sp: High (2/2); Su: Mod. (1/2); Fa: Low (1/2)	Wi: Low (2/2); Sp: Low (2/2); Su: Low (1/2); Fa: Low (1/2)
**Suffolk**	1	3	High (3/3)	Wi: Low (3/3); Sp: Low (3/3); Su: Low (3/3); Fa: Low (3/3)	Wi: Mod. (2/3); Sp: Low (3/3); Su: Low (3/3); Fa: Low (3/3)	Wi: Low (3/3); Sp: Mod. (2/3); Su: Low (2/3); Fa: Low (2/3)
	2	2	Low (1/2)	Wi: Low (1/2); Sp: Low (2/2); Su: Low (2/2); Fa: Low (2/2)	Wi: High (2/2); Sp: Low (1/2); Su: Low (1/2); Fa: Mod. (1/2)	Wi: Mod. (1/2); Sp: Mod. (1/2); Su: Mod. (2/2); Fa: Low (2/2)
	3	1	Mod. (1/1)	Wi: Mod. (1/1); Sp: High (1/1); Su: High (1/1); Fa: High (1/1)	Wi: Low (1/1); Sp: Mod. (1/1); Su: Mod. (1/1); Fa: Low (1/1)	Wi: Low (1/1); Sp: Low (1/1); Su: Low (1/1); Fa: Low (1/1)
	4	7	Low (7/7)	Wi: Low (3/7); Sp: Mod. (5/7); Su: Mod. (7/7); Fa: Mod. (5/7)	Wi: Mod. (5/7); Sp: Mod. (6/7); Su: Mod. (6/7); Fa: Mod. (5/7)	Wi: High (4/7); Sp: High (4/7); Su: High (4/7); Fa: High (5/7)

1 Categories for elevation were defined using fixed thresholds (low <500 m, moderate 500–1,000 m, high >1,000 m).

2 Values in parentheses indicate the number of flocks in the most common category relative to the total number of flocks in that cluster.

3 Categories for seasonal climate variables (CCI, precipitation, soil moisture) were defined using global tercile thresholds derived from the distribution across all flocks; seasons were defined as Wi = winter (December 1–February 28/29), Sp = spring (March 1–May 31), Su = summer (June 1–August 31), and Fa = fall (September 1–November 30).

### Management-based clustering (management clusters)

Management clusters reflected differences in husbandry practices amongst flocks within breeds. Based on their survey responses, producers made management decisions that accounted for both the characteristics of their breed and the constraints of their production environments.

#### Katahdin

Management-based clustering of Katahdin flocks revealed marked differences in parasite management, culling practices, feeding strategies, flock size, and producer-reported ground surface moisture and flooring conditions (Figure 2, middle panel).

Parasite management primarily distinguished management clusters 1 and 3. Management cluster 1 consisted of 18 flocks, none of which dewormed. In contrast, management cluster 3 was defined by 29 flocks with active parasite control, including a reliance on macrocyclic lactone drugs with frequent use of combinations of deworming products. Anthelmintic treatment focused on lambs, typically used when first deemed necessary, with ewes dewormed rarely or not at all. Flocks in cluster 3 also avoided purchasing replacement ewes, which was uncommon in the other management clusters in Katahdin.

Parasite-related strategies were less influential in defining management clusters 2, 4, and 5. Instead, they were distinguished based on culling and feeding practices, flock size, or ground surface moisture and flooring conditions. Management cluster 2 represented a single flock that culled ewes due to udder health and reproductive problems, moved animals to new locations during the production season, grazed a large land base (> 405 ha), and retained larger numbers of ewe lambs as replacements (> 250). Management cluster 4 also consisted of a single flock that did not graze instead relying on prepared diets. This flock culled ewes based on age, udder health, and unfavorable estimated breeding values (EBV). Management cluster 5 likewise consisted of a single flock and was characterized by a location with wet ground conditions, late-summer or fall shearing, and larger ram purchases (> 10 rams). Note this was one of only a few Katahdin flocks that sheared. This flock also culled ewes based on udder health, thin body condition, and illness.

#### Polypay

Unlike Katahdin, polypay clusters were shaped more by health- and culling-related practices than by parasite management. Management-based clustering of Polypay flocks was characterized by differences in culling practices, feeding and neonatal-care strategies, replacement decisions, and flock infrastructure (Figure 3, middle panel).

Management cluster 1 was defined by eight flocks that did not deworm. Beyond no parasite control, these flocks reported low mastitis-related culling (<5% of ewes). In general, they did not provide supplemental feed during cold weather.

Management cluster 2 consisted of a single flock that culled ewes for foot and leg problems, thin or unthrifty body condition, and less desirable EBV. This flock purchased some replacement ewes, which were dewormed before mixing with the rest of flock. It also reported not providing protein or energy supplements.

Management cluster 3 also consisted of a single flock that operated on a grazing land base of 201 to 405 ha. This flock, which reported more than 250 ewes lambed annually, retained a relatively large number of ewe lambs as replacements (101 to 250), and reported seeking veterinary intervention for GIN issues within the past year. Culling decisions were primarily based on udder health problems, thin or unthrifty body condition, and illness.

Management cluster 4 consisted of six flocks that provided extra care for weak or triplet lambs, either through hand feeding or in combination with natural nursing. These flocks did not purchase outside replacement ewes. They operated on relatively small land areas (4 to 20 ha) and reported routine deworming as part of their parasite-control management.

Management cluster 5 included four flocks that reported low expenditures on dewormers (< $100 annually), and no mastitis-related culling or issues with thin or unthrifty ewes. These flocks relied on simple housing structures with three or fewer walls (such as loafing or transportable sheds) and typically moved ewes with lambs to pasture shortly after birth. They exclusively used irrigated pastures for grazing rather than cropland or a mixture of cropland and grassland and provided no supplementary feeding.

Management cluster 6 consisted of a single flock managed entirely on pasture, with no housing structures available for lambing. This operation reported severe udder health challenges: 10% to 30% of lambing ewes had clinical mastitis and over 30% were culled primarily due to mastitis. This flock incurred between $300 and $500 expenses to treat mastitis for between 51 and 100 ewes lambing annually.

#### Rambouillet and Targhee

Management-based clustering of Rambouillet–Targhee flocks revealed variation in lambing practices, parasite-control strategies, culling decisions, and in housing provided (Figure 4, middle panel). Producers reported the broadest range of management systems in this breed-type.

Management cluster 1 was defined by a single flock that turned lambs out to pasture shortly after birth and reported culling decisions driven by temperament, genetic merit, and economic concerns. This flock indicated that 16% to 30% of lambs were considered poor doers due to GIN parasitism, did not provide supplemental vitamins or minerals to ewes on pasture or rangeland, and transferred animals off the home site for grazing, typically for 4 to 6 mo.

Management cluster 2 represented the largest group of Rambouillet–Targhee flocks. These six flocks did not manage first-lambing ewes separately from the main flock, and typically turned-out lambs to pasture before 30 d of age. They reported no transfer of animals to other sites.

Management cluster 3 included two flocks that did not place lambs on pasture. Instead, lambs were confined until weaning. When grazing was practiced in ewes, it extended 3 to 6 mo annually. Ram lambs were generally left intact, and flocks reported close management during lambing, with lambing areas checked every 4 h. Lambs were also weighed three times during their first year of life, reflecting a relatively high degree of monitoring and record-keeping.

Management cluster 4 was defined by a single flock with a structured parasite control regime. This flock alternated dewormers within the same grazing season, treated lambs prior to weaning for parasites, and spent $100 to $300 annually on deworming products. Culling of ewes was based on age, udder health, and thin or unthrifty body conditions. Winter housing was more protective, with animals kept in fully enclosed structures.

Management cluster 5 consisted of two flocks that reported higher expenditures on deworming products ($300 to $500 annually) and culled ewes primarily for age, teeth, and udder health. Feeding practices supported weak or triplet lambs, with both hand-feeding and nursing strategies used. These flocks avoided macrocyclic lactone dewormers and administered first parasite treatments to lambs only once before marketing or first breeding.

Lastly, management cluster 6 was represented by a single flock. It did not deworm and marked all related questions to GIN control as “not applicable.”

#### Suffolk

Management-based clustering of Suffolk flocks revealed dissimilarities in flock sizes, housing structures, feeding strategies, and culling criteria (Figure 5, middle panel). In this breed, the most striking difference among flocks was between confinement and grazing systems, coupled with variation in the scale of the operation.

Management cluster 1 was defined by a single flock that was distinct because it kept lambs confined in a barn for more than 90 d before turnout into a larger drylot, culled heavily for udder health, genetic merit, and illness, and used prepared diets. This flock also answered “not applicable” on grazing questions, suggesting a strictly confinement-based system.

Management cluster 2 included three flocks that operated on small acreages (4 to 20 ha), retained all ram lambs intact, and housed their animals in fully enclosed structures during the winter. These flocks also reported not transferring animals to other sites.

Management cluster 3 consisted of two flocks that turned ewes and lambs to pasture relatively early (< 30 d age) and operated on moderate acreages (21 to 40 ha). Parasite management was more structured amongst flocks in cluster 3, with alternating use of deworming products during a grazing season with their first anthelmintic treatments typically administered around weaning. Furthermore, these producers indicated that fewer than 5% of lambs were poor doers due to GIN infection.

Management cluster 4 was defined by three flocks that did not manage first-lambing ewes separately from mature ewes and did not select for improving udder health. These flocks reported moderate annual expenditure on deworming products ($300 to $500). They also transferred animals to other sites, typically by truck.

All three flocks in management cluster 5 grazed 1 to 3 mo per yr and retained 16 to 30 ewe lambs as replacements, with two flocks reporting lambing 51 to 100 ewes annually and one flock reporting lambing 31 to 50 ewes annually. Summer housing was generally open-sided sheds, defined as structures with three or fewer walls. Land sizes tended to be small, with two flocks reporting operations with less than 4 ha. However, this cluster also included one operation of substantially larger size (41 to 200 ha). Weak and triplet lambs were provided with additional care; hand-feeding was reported as one husbandry practice.

Management cluster 6 was a single very large-scale operation. The flock was grazed on over 405 ha with more than 250 ewes bred and more than 250 ewe lambs retained as replacements. Ram lamb management was distinctive, with more than half castrated. Lambs were expected to nurse naturally, with no supplemental handfeeding reported.

#### Across breeds

The factors separating management clusters differed by breed. In Katahdin, parasite-control strategy (no deworming vs. targeted [selective] deworming and use of combination of deworming products) primarily separated flocks into clusters. In Polypay, clusters were distinguished by health and culling practices (eg udder health, structural soundness) and differences in flock infrastructure. In Suffolk, cluster assignments reflected differences in production systems (grazing vs. confinement) and operation size. Rambouillet–Targhee showed the broadest range of husbandry practices, from confinement-oriented to extensive grazing. No single management practice dominated flocks’ separation into clusters in these fine-wool breeds. Instead, differences among flocks in parasite control, health management, and feeding practices equally contributed to distinguishing management clusters.

### Combined climate- and management-based clustering (eco-management clusters)

The integration of climate and management data using FAMD, followed by LDA, generated distinct clustering patterns within each breed. These eco-management clusters reflected flocks that shared similar climatic conditions and husbandry practices.

#### Katahdin

Katahdin flocks were classified into six eco-management clusters (Figure 2, bottom panel; Table 7). These clusters ranged from high elevation, cold, dry year-round environments with no GIN-related losses reported (eco-management cluster 2) to year-round wet environments characterized by high precipitation and soil moisture and substantial GIN-associated losses (eco-management cluster 6). In general, vulnerability to GIN increased in the wettest environments. Shearing was rare, consistent with Katahdin sheep naturally shedding their hair coat. Where shearing occurred, it was observed in flocks experiencing persistently wet conditions, including both high- and low-elevation locales.

**Table 7:** Summary of eco-management clusters defined for the Katahdin breed.

Eco-management cluster		Elevation^1^^,^^2^	Seasonal climate variables				GIN infection	Shearing^2^	Ram use	Annual weighing frequency	Deworm cost	Parasite resistance selection strategy	Culling focus
Category	No. of flocks		Season	CCI^2^^,^^3^	Precipitation^2^^,^^3^	Soil moisture^2^^,^^3^							
**1**	2	High (1/2)	Wi	High (2/2)	Low (2/2)	Low (2/2)	None reported	Not shorn	3–5	3×	None	50% FEC EBV and phenotype, 50% no response	Age, repro., body condition
			Sp	High (2/2)	Low (2/2)	Low (2/2)							
			Su	High (2/2)	Low (2/2)	Low (2/2)							
			Fa	High (2/2)	Low (2/2)	Low (2/2)							
**2**	1	High (1/1)	Wi	Low (1/1)	Low (1/1)	Low (1/1)	None reported	Shorn in Sp.	>10	>3×	None	None reported	Udder, repro., body condition
			Sp	Low (1/1)	Low (1/1)	Low (1/1)							
			Su	Low (1/1)	Low (1/1)	Low (1/1)							
			Fa	Low (1/1)	Low (1/1)	Low (1/1)							
**3**	16	Low (16/16)	Wi	High (6/16)	High (9/16)	High (8/16)	81% none, 19% <5%	Not shorn (94%), 6% shorn in Sp.	3–5 (50%), 1–2 (38%), 6–10 (12%)	Mixed (1–3×, >3×)	None	50% combo, 25% EBV, 25% phenotype, 10% no response	Udder, repro., body condition
			Sp	High (7/16)	High (12/16)	High (8/16)							
			Su	High (7/16)	High (8/16)	High (9/16)							
			Fa	High (7/16)	High (9/16)	High (10/16)							
**4**	29	Low (27/29)	Wi	High (16/29)	High (18/29)	High (18/29)	28% none, 48% <5%, 24% 5–30%	Not shorn (97%), 3% shorn in both Sp. and late Su. and Fa.	3–5 (52%), 1–2 (28%), 6–10 (14%), >10 (7%)	>3 × (59%), others varied	<$100–>$500	62% combo, 25% EBV, 3% phenotype	Repro., udder, body condition
			Sp	High (14/29)	High (16/29)	High (16/29)							
			Su	Low (12/29)	High (16/29)	High (15/29)							
			Fa	High (14/29)	High (18/29)	Mod. (14/29)							
**5**	1	Low (1/1)	Wi	High (1/1)	Mod. (1/1)	Mod. (1/1)	None reported	Not shorn	3–5	2×	$100–300	Phenotype or EBV	Age, udder, genetics
			Sp	High (1/1)	High (1/1)	Mod. (1/1)							
			Su	High (1/1)	High (1/1)	High (1/1)							
			Fa	High (1/1)	Mod. (1/1)	High (1/1)							
**6**	1	Low (1/1)	Wi	High (1/1)	High (1/1)	High (1/1)	>30% losses	Shorn (late Su. and Fa.)	>10	>3×	>$500	Combo	Udder, body condition, illness
			Sp	High (1/1)	High (1/1)	High (1/1)							
			Su	Mod. (1/1)	High (1/1)	High (1/1)							
			Fa	Mod. (1/1)	High (1/1)	High (1/1)							

1 Categories for elevation were defined using fixed thresholds (low <500 m, moderate 500–1,000 m, high >1,000 m).

2 Values in parentheses indicate the number of flocks in the most common category relative to the total number of flocks in that cluster.

3 Categories for seasonal climate variables (CCI, precipitation, soil moisture) were defined using global tercile thresholds derived from the distribution across all flocks; seasons were defined as Wi = winter (December 1–February 28/29), Sp = spring (March 1–May 31), Su = summer (June 1–August 31), and Fa = fall (September 1–November 30).

Ram use varied but most flocks maintained small groups of rams (3 to 5), with a few large flocks that used more than 10 rams annually. Katahdin producers weighed their animals at least once annually, although most at least twice. Deworming costs differed sharply: flocks in drier environments often reported no expenditure, whereas those in wetter regions generally incurred moderate to high costs. Still, some flocks located in wetter climates reported no such expenditures. Culling practices were broadly consistent amongst flocks, focused on udder health, reproductive performance, and body condition, with some flocks also considering age, genetics, or illness. Strategies to improve parasite resistance differed among flocks. Some producers based their selection decisions on a combination of fecal egg count (FEC) EBV and phenotypic performance; others used only one of these criteria, while still others reported no formal strategy for reducing susceptibility to GIN.

Comparisons across clustering approaches also illustrate how flocks grouped together under climate-based clustering can separate when management practices are incorporated. For example, three Katahdin flocks grouped together in eco cluster 1 due to broadly similar seasonal precipitation and soil moisture profiles. However, when management variables were also incorporated, one of these flocks separated into eco-management cluster 2, while the other two remained together in eco-management cluster 1. This re-assignment reflected the distinct shearing, ram use, GIN control, and culling strategies of the now separated flock along with its lower seasonal CCI values.

#### Polypay

Eco-management clustering of Polypay flocks identified six distinct groupings (Figure 3, bottom panel; Table 8). These clusters spanned environments from dry, low-elevation systems with consistently low precipitation and soil moisture (eco-management clusters 1 and 4) to wetter, low-elevation environments with moderate to high precipitation and soil moisture (eco-management clusters 6). Mastitis prevalence was generally low, though burdens increased under wetter conditions, particularly in eco-management cluster 6 where 10% to 30% of ewes were affected and more than 30% were culled for mastitis.

**Table 8:** Summary of eco-management clusters defined for the Polypay breed.

Eco-management cluster		Elevation^1^^,^^2^	Seasonal climate variables				Mastitis (prevalence, culling, cost)	Deworming	Culling focus	Grazing management	Summer housing	Flock size
Category	No. of flocks		Season	CCI^2^^,^^3^	Precipitation^2^^,^^3^	Soil moisture^2^^,^^3^						
**1**	5	Low (4/5)	Wi	Low (5/5)	Low (5/5)	Low (5/5)	Prevalence <5%, culling <5%, cost <$100	None or minimal	Udder, repro., body condition, foot and leg problems	Pasture-only (40%), mixed (pasture/cropland) (40%), cropland (20%)	No permanent housing (80%), permanent structure (20%)	16–100 ewe lambs, one >250
			Sp	Low (3/5)	Low (5/5)	Low (5/5)						
			Su	Mod. (3/5)	Low (4/5)	Low (4/5)						
			Fa	Low (3/5)	Low (5/5)	Low (4/5)						
**2**	4	Low (4/4)	Wi	Low (3/4)	Low (4/4)	Low (2/4)	Prevalence <10% (most <5%), culling <5%, cost $100–$300 (some <$100)	Mostly no dewormers (75%)	Udder, repro., body condition	No pasture, grazing not applicable	No housing (50%), permanent structure (50%)	16–100 ewe lambs (most 51–100, some smaller)
			Sp	Mod. (4/4)	Low (3/4)	Low (3/4)						
			Su	High (3/4)	Mod. (2/4)	Low (2/4)						
			Fa	Mod. (4/4)	Low (2/4)	Low (2/4)						
**3**	1	Mod. (1/1)	Wi	Mod. (1/1)	Low (1/1)	Low (1/1)	Prevalence 5–10%, culling <5%, cost $100–300	Combination of dewormers	Udder, body condition, illness	Pasture-only	No summer housing (100%)	101–250 ewe lambs
			Sp	High (1/1)	Mod. (1/1)	Low (1/1)						
			Su	High (1/1)	Mod. (1/1)	Low (1/1)						
			Fa	High (1/1)	Low (1/1)	Low (1/1)						
**4**	3	Low (2/3)	Wi	Low (2/3)	Low (2/3)	Low (2/3)	Prevalence 0% to <5%, culling none or <5%, cost <$100	Alternating dewormers	Udder, repro., temperament, illness	Pasture-only	No housing (67%), loafing shed (33%)	<31 ewe lambs
			Sp	Low (2/3)	Low (3/3)	Low (3/3)						
			Su	Low (1/3)	Low (3/3)	Low (2/3)						
			Fa	Low (2/3)	Low (3/3)	Low (2/3)						
**5**	7	Low (5/7)	Wi	Mod. (4/7)	Mod. (5/7)	Mod. (6/7)	Prevalence usually <5% (some 5–10%), culling <10%, cost $100–$300 or <$100	Mixed: alternating, single, combination	Udder, repro., body condition, genetics	Pasture-only (57%), mixed (43%)	No housing (57%), forested (29%), barn (14%)	16–30 ewe lambs (most), some 31–50 or 51–100
			Sp	Mod. (5/7)	Mod. (5/7)	Mod. (5/7)						
			Su	Low (3/7)	Mod. (4/7)	Mod. (4/7)						
			Fa	Mod. (5/7)	Mod. (3/7)	Low (4/7)						
**6**	1	Low (1/1)	Wi	High (1/1)	High (1/1)	Mod. (1/1)	Prevalence 10–30%, culling >30%, cost $300–$500	Combination of dewormers	Udder, repro., genetics	Mixed	Forested area (100%)	16–30 ewe lambs
			Sp	Mod. (1/1)	Mod. (1/1)	High (1/1)						
			Su	Low (1/1)	Mod. (1/1)	Mod. (1/1)						
			Fa	Mod. (1/1)	Mod. (1/1)	Mod. (1/1)						

1 Categories for elevation were defined using fixed thresholds (low <500 m, moderate 500–1,000 m, high >1,000 m).

2 Values in parentheses indicate the number of flocks in the most common category relative to the total number of flocks in that cluster.

3 Categories for seasonal climate variables (CCI, precipitation, soil moisture) were defined using global tercile thresholds derived from the distribution across all flocks; seasons were defined as Wi = winter (December 1–February 28/29), Sp = spring (March 1–May 31), Su = summer (June 1–August 31), and Fa = fall (September 1–November 30).

Parasite control strategies differed amongst flocks. Operations located in the driest environments, where precipitation and soil moisture were low year-round (eco-management clusters 1 and 4), reported either minimal deworming or the use of alternating anthelmintic products. Conversely, operations in clusters experiencing more seasonal or consistently higher moisture (eco-management clusters 3, 5, and 6) more commonly alternated amongst or combined anthelmintics. The exception was eco-management cluster 2, in which most flocks did not deworm despite moderate summer precipitation.

Culling decisions were consistently based on udder health and reproduction, but flocks in some clusters also considered genetics (EBV), body condition, foot and leg soundness, dentition, or illness as culling criteria. Feeding systems ranged from pasture-only to, in some flocks, a mix of pasture and residual grazing of cropland. In summer, housing was typically minimal or absent, although some operations reported using barns, loafing sheds, or shaded forested areas to mitigate climatic conditions.

#### Rambouillet and Targhee

Rambouillet and Targhee flocks were grouped into six eco-management clusters (Figure 4, bottom panel; Table 9). These clusters spanned environments from cooler, generally drier year-round conditions at higher elevations (eco-management clusters 1 and 3) to moderate elevation systems that were also dry in all seasons but with milder (eco-management cluster 2) to warmer temperatures (eco-management cluster 5) year-round. The climatic conditions of flocks at lower elevations differed more appreciably: eco-management cluster 4 experienced cool winters and increasingly wet soils moving into summer and fall, whereas eco-management cluster 6 was warm year-round with higher spring rainfall.

**Table 9:** Summary of eco-management clusters defined for the Rambouillet–Targhee breeds.

Eco-management cluster		Elevation^1^^,^^2^	Seasonal climate variables				Deworming and cost for treatment	Culling focus	Winter housing	Ram purchase	Flock size
Category	No. of flocks		Season	CCI^2^^,^^3^	Precipitation^2^^,^^3^	Soil moisture^2^^,^^3^					
**1**	1	High (1/1)	Wi	Low (1/1)	Mod. (1/1)	Low (1/1)	No deworming, <$100 treatment	Udder, repro., body condition	None	1–2	Very large: >250 ewes, >250 ewe lambs
			Sp	Low (1/1)	Low (1/1)	Mod. (1/1)					
			Su	Low (1/1)	Low (1/1)	Low (1/1)					
			Fa	Low (1/1)	Low (1/1)	Low (1/1)					
**2**	5	Mod. (3/5)	Wi	Mod. (3/5)	Low (5/5)	Low (5/5)	Mixed (combo 40%, alternating 40%, single 20%), treatments weaning/needed, spending <$100–$500	Age, udder, repro., teeth, body condition	Minimal (none 60%, barn 20%, 3-sided shed 20%)	3–5 (60%), 1–2 (20%), 6–10 (20%)	Large: >250 ewes (80%), replacements 51–>250
			Sp	Mod. (3/5)	Low (5/5)	Low (5/5)					
			Su	Mod. (3/5)	Low (5/5)	Low (5/5)					
			Fa	Low (4/5)	Low (5/5)	Low (5/5)					
**3**	2	High (1/2)	Wi	Low (2/2)	Low (2/2)	Low (2/2)	Mixed (combo 50% or alternating 50%), treat at turnout/needed, <$100	Age, udder, repro., temperament	None (100%)	1–2 (100%)	Medium: 101–250 ewes, replacements 16–50
			Sp	Low (1/2)	Low (2/2)	Low (2/2)					
			Su	Low (1/2)	Low (1/2)	Low (1/2)					
			Fa	Low (2/2)	Low (2/2)	Low (1/2)					
**4**	1	Low (1/1)	Wi	Low (1/1)	Low (1/1)	Mod. (1/1)	Single dewormer, rare use, <$100	Age, repro., genetics	3-sided shed	1–2	Small: 51–100 ewes, <16 replacements
			Sp	Low (1/1)	Mod. (1/1)	Mod. (1/1)					
			Su	Mod. (1/1)	High (1/1)	High (1/1)					
			Fa	Low (1/1)	Mod. (1/1)	High (1/1)					
**5**	2	Mod. (2/2)	Wi	High (2/2)	Low (2/2)	Low (1/2)	Combo dewormers, treat at turnout, <$100	Age, teeth, economic factors, udder, repro.	None (50%), forested (50%)	1–2	Medium–large: 101–250 or >250 ewes, replacements 16–250
			Sp	High (2/2)	Low (2/2)	Low (2/2)					
			Su	High (2/2)	Low (2/2)	Low (2/2)					
			Fa	High (2/2)	Low (2/2)	Mod. (2/2)					
**6**	2	Low (2/2)	Wi	High (2/2)	Low (1/2)	Low (2/2)	Combo dewormers, treat when needed, <$100	Repro., body condition, genetics	None (100%)	1–2 (50%), none (50%)	Variable: 16–250 ewes, replacements 16–50
			Sp	High (2/2)	High (2/2)	Low (2/2)					
			Su	High (2/2)	Mod. (1/2)	Low (1/2)					
			Fa	High (2/2)	Low (1/2)	Low (1/2)					

1 Categories for elevation were defined using fixed thresholds (low <500 m, moderate 500–1,000 m, high >1,000 m).

2 Values in parentheses indicate the number of flocks in the most common category relative to the total number of flocks in that cluster.

3 Categories for seasonal climate variables (CCI, precipitation, soil moisture) were defined using global tercile thresholds derived from the distribution across all flocks; seasons were defined as Wi = winter (December 1–February 28/29), Sp = spring (March 1–May 31), Su = summer (June 1–August 31), and Fa = fall (September 1–November 30).

Management strategies reflected differences in temperatures and rainfall patterns amongst the Rambouillet and Targhee flocks. Deworming strategies varied across clusters. The single flock in eco-management cluster 1 did not treat for GIN parasites, whereas the single flock in eco-management cluster 4 treated only rarely. Flocks in the other clusters used a variety of approaches, including combining and alternating anthelmintic products. These flocks treated lambs at weaning (eco-management cluster 2), when first turned out to pasture (eco-management clusters 3 and 5), or when signs of parasitism arose (eco-management cluster 6). Annual costs for parasite treatments were typically low (less than $100), though one flock in eco-management cluster 2 reported spending up to $500 annually.

Culling decisions often combined age with udder and reproductive soundness. However, other criteria were also used including thin body condition, dentition problems, less desirable genetics (EBV), temperament, and economic considerations (eg drought, market conditions).

Winter housing was minimal. Most flocks had no permanent structures available, although some used three-sided sheds, enclosed barns, or forested areas. Flock sizes were larger in eco-management clusters at higher and moderate elevations (> 250 ewes in general), while clusters located at lower elevations tended to maintain smaller operations. Ram purchase was usually limited to 1 to 5 sires; some flocks, though, reported larger numbers of rams purchased or even no purchases.

#### Suffolk

Eco-management clustering of Suffolk flocks identified four distinct groupings (Figure 5, bottom panel; Table 10). These clusters included flocks located at high and moderate elevations and that either had cooler drier (eco-management clusters 1 and 2) or warmer wetter (eco-management cluster 4) conditions to low-elevation environments with moderate moisture levels (eco-management cluster 3).

**Table 10:** Summary of eco-management clusters defined for the Suffolk breed.

Eco-management cluster		Elevation^1^^,^^2^	Seasonal climate variables				Castration practices	Pasture use (lambs)	Feeding system	Grazing duration	Summer housing	Ram use	Culling focus	Flock size
Category	No. of flocks		Season	CCI^2^^,^^3^	Precipitation^2^^,^^3^	Soil moisture^2^^,^^3^								
**1**	2	High (1/2)	Wi	Low (1/2)	Mod. (2/2)	High (1/2)	>50% castrated (50%), none (50%)	None (50%), 30–60 d (50%)	Pasture and prepared diets	1–3 mo (50%), >6 mo (50%)	No structure (50%), 3-sided shed (50%)	3–5 (50%), >10 (50%)	Udder, repro, body condition	Ewes: 51–100 or >250 (50% each), ewe lambs: 16–30, >250
			Sp	Low (1/2)	Low (1/2)	High (1/2)								
			Su	Low (1/2)	Low (1/2)	High (1/2)								
			Fa	Low (1/2)	Low (1/2)	High (1/2)								
**2**	3	Mod. (2/3)	Wi	Low (2/3)	High (1/3)	Low (2/3)	<25% castrated (100%)	<30 d (67%), none (33%)	Pasture and prepared diets	3–6 mo (67%), >6 mo (33%)	No structure (100%)	1–2 (33%), 3–5 (33%), 6–10 (33%)	Repro, udder, body condition	Ewes: 16–250, ewe lambs: <16 (67%), 51–100 (33%)
			Sp	Low (3/3)	Low (3/3)	High (1/3)								
			Su	Low (3/3)	Low (3/3)	Mod. (2/3)								
			Fa	Low (3/3)	Low (2/3)	Low (2/3)								
**3**	7	Low (6/7)	Wi	Low (4/7)	Low (3/7)	Mod. (4/7)	None (71%), <25% (14%), 25–50% (14%)	None (86%), 30–60 d (14%)	Pasture and prepared diets	1–3 mo (29%), 3–6 mo (43%), >6 mo (29%)	No structure (72%), forest (14%), 3-walled shed (14%)	3–5 (86%), 1–2 (14%)	Udder, repro, body condition	Ewes: 31–100, ewe lambs: <16 (57%), 16–30 (29%), 51–100 (14%)
			Sp	Low (3/7)	Mod. (6/7)	Mod. (4/7)								
			Su	Mod. (5/7)	Mod. (6/7)	Mod. (4/7)								
			Fa	Mod. (4/7)	Low (3/7)	High (3/7)								
**4**	1	High (1/1)	Wi	High (1/1)	Mod. (1/1)	High (1/1)	<25% castrated	>90 d	Prepared diets only	No grazing	Fully enclosed barn	1–2	Udder, genetics, illness	Very small: <16 ewes and ewe lambs
			Sp	Mod. (1/1)	Mod. (1/1)	High (1/1)								
			Su	Mod. (1/1)	Mod. (1/1)	High (1/1)								
			Fa	High (1/1)	Mod. (1/1)	High (1/1)								

1 Categories for elevation were defined using fixed thresholds (low <500 m, moderate 500–1,000 m, high >1,000 m).

2 Values in parentheses indicate the number of flocks in the most common category relative to the total number of flocks in that cluster.

3 Categories for seasonal climate variables (CCI, precipitation, soil moisture) were defined using global tercile thresholds derived from the distribution across all flocks; seasons were defined as Wi = winter (December 1–February 28/29), Sp = spring (March 1–May 31), Su = summer (June 1–August 31), and Fa = fall (September 1–November 30).

Castration practices varied among Suffolk flocks, with eco-management cluster 1 showing a distinctly polarized pattern, in which producers either castrated most of their ram lambs or did not castrate at all. Across the remaining clusters, castration was generally avoided or limited to fewer than 25% of ram lambs.

Feeding and grazing practices also differed across clusters, with eco-management clusters 1, 2, and 3 combining pasture with prepared diets, while eco-management cluster 4 relied exclusively on prepared diets and did not graze. Across clusters 1 through 3, grazing duration for ewes ranged from short (1 to 3 mo) to extended periods (over 6 mo).

Summer housing was minimal across the Suffolk flocks, with most operations providing either no housing or simple structures such as three-sided sheds. The exception was eco-management cluster 4, a single flock that used fully enclosed barns.

Culling decisions consistently focused on udder health, reproduction, and body condition, with genetics and illness noted in some eco-management clusters. Flock sizes spanned from very small operations (< 16 ewes) to larger flocks exceeding 250 ewes. Ram use varied widely, ranging from 1 to 2 sires in the smallest operations to more than 10 sires in larger flocks.

## Discussion

Within-breed differences in climate exposure and management strategies play a critical role in shaping U.S. sheep production systems. While fine-wool (Rambouillet–Targhee), terminal sire (Suffolk), and maternal (Polypay and Katahdin) breeds are typically associated with distinct production goals, substantial heterogeneity in both climatic exposure and management practices also exist within each breed-type. Traditional geographic classifications, therefore, may fail to capture how producers respond to local climatic challenges through breed- and system-specific management decisions.

Integrating climate and management data does not imply that eco-management clustering consistently identifies fundamentally different production environments from those defined by climate or management alone. However, our results reinforce the view that production environments are not defined by geography alone. Instead, producers’ husbandry decisions are in response, at least in part, to the prevailing weather conditions affecting their operations. Nonetheless, the relative contributions of climate and management to the characterization of a flock’s production environment differed across breeds.

These findings are consistent with previous work. For example, Arisman et al. (2023) reported that eco-management clusters explained more variation in traits measured around weaning than climate or management clusters alone. Similarly, Rowan et al. (2021) demonstrated in beef cattle that integrating environmental and management-related variables yields more biologically meaningful classifications of production environments. Our findings extend this logic to the small ruminant sector by showing that even greater explanatory power is possible when producer-reported management practices are considered.

### Multivariate clustering

Multivariate procedures, such as PCA, MCA, and FAMD, are well suited to clustering complex qualitative and quantitative data into a limited number of discrete categories. However, they are inherently exploratory methodologies. The clusters obtained depend on the distance measures used to reduce the dimensionality of multivariate surfaces. We used different multivariate methods and datasets to form eco, management, and eco-management clusters. Therefore, the variation (inertia) explained within a given number of principal components cannot be directly compared amongst our clustering strategies. Less distinct cluster assignments, particularly for management and eco-management clustering, were therefore expected given the high dimensionality and diversity of management practices considered (Aggarwal et al. 2001).

A concern when constructing eco-management clusters using FAMD was the potential of a disproportionate contribution of the management variables, as management data greatly outnumbered climate variables. However, the concordance between eco-management and eco clusters in Katahdin, Polypay, and Rambouillet–Targhee suggests that both data sources contributed meaningfully to cluster formation. In Suffolk, although climate-based clustering demonstrated the strongest cohesion, the lower agreement between eco and eco-management clusters suggests that management decisions may not have been as affected by climatic conditions in this breed. This balance supports the use of FAMD as a practical tool for integrating producer-reported management data with continuous climate variables.

### Cluster assignments

In Katahdin, flocks assigned together to a management-based cluster were also assigned to the same eco-management cluster. This breed was most dispersed geographically amongst those considered in the study. Still, most producers adopted similar management practices despite these varied climates. This perhaps reflects strong common priorities amongst hair sheep producers, such as adopting practices that mitigate GIN parasitism. In Polypay, the same subset of flocks also tended to be assigned to the same management and eco-management clusters. However, in contrast to Katahdin, Polypay flocks located in broadly similar climatic regions—particularly those flocks in Western and Upper Midwestern states—often adopted different husbandry practices. This suggests a weaker alignment between climate and management decisions in this breed.

In Rambouillet–Targhee flocks, assignments to eco-management clusters most closely resembled assignments to eco clusters. This is perhaps unsurprising. Most Rambouillet and Targhee flocks are grazed on large, extensive landscapes. The repertoire of husbandry practices available to a producer is more limited, although differences among flocks particularly in the Intermountain West region were observed. Still, geography and weather conditions play a dominant role in defining production environments in this breed-type.

The least consistent assignment of flocks to clusters was observed in Suffolk, which likely reflects underlying differences in breeding objectives within the breed, including divergent selection toward distinct biological types. As noted by Kuehn et al. (2009), Suffolk flocks in the U.S. have been selected along two main trajectories: one emphasizing traditional terminal-sire characteristics such as larger mature size, and another favoring more moderate mature size with greater emphasis on muscling. These divergent breeding objectives are also associated with reduced exchange of breeding stock among flocks, which can limit connectedness and contribute to the separation of flocks into distinct clusters even within similar geographic regions. This likely coincides with producers adopting different husbandry practices even under comparable climatic conditions, with little correspondence between geographic location and management practices. This lack of alignment underscores that combining climate and management practices into eco-management clusters is particularly important in this breed to more accurately capture a flock’s production environment.

Taken together, the way in which producers tailored their management practices to mitigate the prevailing weather conditions affecting their flocks differed among breeds. In part, this reflects different breeds being predominant in different regions. In some breeds, such as Rambouillet and Targhee, the strategies used are often shared within climatic regions. In other breeds, such as Suffolk, different approaches are adopted even within similar climates. Still, by combining information about climates and husbandry practices, flocks can be bundled into categories of production environments that delineate their similarities and dissimilarities. Doing so predicates identifying possible G × E, or sensitivities, that may affect selection programs within individual breeds.

More broadly, our results align with research that has considered genotype-by-environment-by-management interactions, demonstrating that management mediates genetic expression across environments (Carrick and van der Werf 2005; O’Neill and Swain 2015). Recent work by Santana et al. (2025) further supports that observation: farm-level management descriptors provide explanatory power beyond conventional contemporary group effects when combined with environmental information. In this context, eco-management clusters provide a conceptually robust framework for defining production environments based on both climate and management, rather than geography alone, thereby supporting more meaningful comparisons among animals in genetic evaluations.

### Interplay between climate and producer decision-making

The importance of these breed-specific and climate-linked management patterns is underscored by the geographic distribution of the U.S. sheep industry. As of January 1, 2025, the total U.S. sheep and lamb inventory was estimated at 5.05 million animals. The Intermountain West region accounted for the largest share (32.4%), followed by the Midwest (23.8%), South (21.8%), West Coast (17.3%), and Northeast (4.7%) (USDA-National Agricultural Statistics Service 2025). While flocks surveyed in the present study were represented across all major U.S. production regions, the distribution did not fully mirror patterns of the national inventory of sheep numbers. For example, the Midwest was proportionally more represented, whereas the Intermountain West and West Coast were comparatively underrepresented relative to national estimates. These differences should be considered when interpreting the broader applicability of the findings, as they may partly reflect variation in the adoption of NSIP across production systems. More extensive operations particularly, which are more common in the Intermountain West, may face greater logistical challenges in performance recording, potentially leading to lower representation in NSIP-based datasets. Nonetheless, eco-management clustering provides a framework for capturing how producers operating within these dominant regions adapt management strategies to local climatic constraints, rather than assuming uniform production systems within broad regions.

Stewart et al. (2020) surveyed 25 sheep operations in the Intermountain West and reported large differences in grazing and parasite control strategies, despite all flocks operating within the same semi-arid climate. Notably, high GIN parasite burdens were associated with irrigated pasture systems, and widespread anthelmintic resistance was documented across multiple dewormer classes. These findings support the idea that producer-driven decisions, such as grazing system design, irrigation use, and treatment protocols, can significantly reshape the biological stressors affecting flocks, independent of regional climate. Our results align with this observation, demonstrating that meaningful management variation exists even among flocks exposed to similar climatic conditions.

Parasite control and nutritional strategies varied among eco-management clusters. Flocks in wetter, warmer regions tended to apply more intensive parasite control, while those in drier climates more often relied on genetic resistance with minimal use of anthelmintics. These differences suggest that parasite management strategies are closely linked to prevailing local climatic conditions. Nutritional differences were also evident. Some flocks provided supplements while others did not, likely reflecting differences in forage availability. Such differences were also observed by Cannas et al. (2019), who argued that feeding and ration balancing must be customized to the production system. That includes accounting for expected animal dry matter intake and the availability, quality, and utilization of forage resources, as well as housing and other management constraints. Together, these findings underscore that management choices remain critical to mitigating biological stressors even when climatic exposure is similar.

The way producers balanced other management factors, such as udder health and culling strategies, also varied across eco-management clusters. Rambouillet–Targhee producers consistently reported selecting for udder health yet rarely cited udder problems as a primary culling reason. This suggests that, although udder health is considered in selection decisions, producers may apply less stringent thresholds when making culling decisions.

### Limitations

The survey was distributed to members of a structured breeding network (NSIP) and was completed voluntarily; it therefore may not fully represent the broader U.S. sheep industry. Cluster profiles are sensitive to the choice and framing of variables (Tremblay et al. 2016), emphasizing the importance of careful survey design and validation against external performance criteria. Both were explicitly addressed in the development and evaluation of the survey instrument used in this study (Yazar Gunes et al. 2026). In addition, elevation was derived from a single georeferenced location per flock, which may not fully capture within-ranch variation, particularly for large-scale operations, such as those common in Rambouillet and Targhee flocks. Finally, increasing the resolution of environmental classification may result in clusters containing very few flocks, which can reduce the robustness and practical utility of such classifications.

### Implications

Our refined classification of production environments has potential implications for U.S. sheep genetic evaluations, particularly for producers engaged in NSIP. By integrating climate and management information, eco-management clustering provides a framework for improving the identification of G × E allowing for better alignment of genetic evaluations with the conditions under which animals are raised. Even producers not enrolled in NSIP may benefit from this framework. By considering the characteristics of eco-management clusters, they can identify flocks with similar climatic and management conditions to their own. Doing so may facilitate their own marketing and breeding programs.

The implementation of eco-management clustering will require the collection of management data from producers in a manner that is practical and scalable, without imposing undue burden on their time. In this context, streamlined data collection approaches, such as concise and clearly structured surveys, may help minimize potential bias in producer responses. Climatic data on the other hand can be derived directly from geographic locations without additional input from producers.

Going forward, a streamlined approach that captures a small set of biologically meaningful management descriptors—such as parasite control strategy, lambing environment, feeding system and culling criteria—may offer a balance between precision and practicality. Because farm management practices evolve over time, periodic reassessment will be necessary for eco-management clusters to remain representative. However, such updates can be targeted and infrequent (eg every 3 to 5 yr or following major operational changes), thereby maintaining their value without imposing undue burden on producers.

## Conclusions

Eco-management clustering provides a structure for defining sheep production environments in the United States that reflects variation in both climate and producer decision-making. By integrating climate variables with producer-reported management practices, we captured dimensions of environmental heterogeneity that neither data source could fully describe alone. The resulting classifications highlight differences in management strategies adopted by producers operating in similar or overlapping climatic conditions. They also illustrated those operational decisions, such as parasite control, feeding strategies, and culling policies, play a central role in shaping the environments in which animals express their genetic potential.

Incorporating eco-management clusters into national evaluation systems has the potential to reduce environmental bias, improve the detection of G × E, and enhance the accuracy of selection decisions. Future work should validate the predictive utility of these clusters within national genetic evaluations and across more diverse flocks, ensuring that genetic progress in U.S. sheep populations is not only accelerated but also more resilient and sustainable in the face of climatic and management challenges.

## Supplementary Material

skag239_Supplementary_Data

## Abbreviations

CCI: comprehensive climate index
EBV: estimated breeding values
FAMD: factorial analysis of mixed data
FEC: fecal egg count
G × E: genotype-by-environment interactions
GIN: gastrointestinal nematodes
HCPC: hierarchical clustering on principal components
IRB: Institutional Review Board
LDA: linear discriminant analysis
MCA: multiple correspondence analysis
NASA POWER: NASA Prediction of Worldwide Energy Resource
NSIP: National Sheep Improvement Program
PCA: principal component analysis

## References

[skag239-B1] Aggarwal CC , Hinneburg A, Keim DA. 2001. On the surprising behavior of distance metrics in high dimensional space. In: Proceedings of the 8th International Conference on Database Theory (ICDT); Springer Berlin Heidelberg. London, UK; p. 420–434. 10.1007/3-540-44503-X_27

[skag239-B2] Aluja T , Morineau A, Sanchez G. 2018. Principal component analysis for data science. https://pca4ds.github.io (accessed 9 January 2025).

[skag239-B3] Arisman BC , Burke JM, Morgan JLM, Lewis RM. 2023. Clustering climate and management practices to define environmental challenges affecting gastrointestinal parasitism in Katahdin sheep. J Anim Sci. 101:1–10. 10.1093/jas/skad002 PMC9904178 36610800

[skag239-B4] Atkinson W. 2024. Charting fields and spaces quantitatively: from multiple correspondence analysis to categorical principal components analysis. Qual Quant. 58:829–848. 10.1007/s11135-023-01669-w

[skag239-B5] Becker RA , Wilks AR, Brownrigg R, Minka TP, Deckmyn A. 2023. maps: draw geographical maps [Computer software]. R package version 3.4.3. https://CRAN.R-project.org/package=maps

[skag239-B6] Benaya R , Sibaroni Y, Ihsan AF. 2023. Clustering content types and user roles based on tweet text using K-medoids partitioning. JoSYC. 4:749–756. 10.47065/josyc.v4i4.3751

[skag239-B7] Cannas A , Tedeschi LO, Atzori AS, Lunesu MF. 2019. How can nutrition models increase the production efficiency of sheep and goat operations? Anim Front. 9:33–44. 10.1093/af/vfz005 PMC6952016 32002249

[skag239-B8] Carrick MJ , van der Werf JHJ. 2005. Environmental variation changes ranking in sire evaluations for wool and health traits in sheep. Proc Assoc Adv Anim Breed Genet. 17:248–251. https://www.aaabg.org/livestocklibrary/2007/carrick248.pdf (accessed 20 February 2025).

[skag239-B9] Kassambara A , Mundt F. 2020. factoextra: extract and visualize the results of multivariate data analyses [Computer software]. R package version 2.0.0. https://CRAN.R-project.org/package=factoextra

[skag239-B10] Kaufman L , Rousseeuw PJ. 1990. Finding groups in data: an introduction to cluster analysis. Wiley.

[skag239-B11] Kuehn LA , Lewis RM, Notter DR. 2009. Connectedness in Targhee and Suffolk flocks participating in the United States National Sheep Improvement Program. J Anim Sci. 87:507–515. 10.2527/jas.2008-1092 18849390

[skag239-B12] Lê S , Josse J, Husson F. 2008. FactoMineR: a package for multivariate analysis. J Stat Softw. 25:1–18. 10.18637/jss.v025.i01

[skag239-B13] Lewis RM , Emmans GC, Simm G. 2006. Describing effects of genetic selection, nutrition, and their interplay in prime lambs using growth and efficiency functions. Aust J Agric Res. 57:707–719. 10.1071/AR05196

[skag239-B14] Mader TL , Johnson LJ, Gaughan JB. 2010. A comprehensive index for assessing environmental stress in animals. J Anim Sci. 88:2153–2165. 10.2527/jas.2009-2586 20118427

[skag239-B15] Maechler M , Rousseeuw P, Struyf A, Hubert M, Hornik K,. 2023. cluster: Cluster analysis basics and extensions. R package version 2.1.8.2. https://CRAN.R-project.org/package=cluster

[skag239-B16] Márquez GC et al 2015. Heterogeneous variances and genetics by environment interactions in genetic evaluation of crossbred lambs. Animal. 9:380–387. 10.1017/S1751731114002717 25407759

[skag239-B17] McManus C et al 2021. Gene bank collection strategies based upon geographic and environmental indicators for beef breeds in the United States of America. Livest Sci. 254:104766. 10.1016/j.livsci.2021.104766

[skag239-B18] NASA POWER. 2024. POWER Data Services. NASA Langley Research Center. https://power.larc.nasa.gov (Accessed 26 February 2024).

[skag239-B19] O’Neill CJ , Swain DL,. 2015. Improving livestock productivity in stressful rangeland environments. Proc. 23rd Int. Congr. Agric. Technol. Transfer (CEA), Asunción, Paraguay.

[skag239-B20] Qualtrics. 2023. Qualtrics XM survey platform. Qualtrics.

[skag239-B21] R Core Team. 2023. R: a language and environment for statistical computing. R Foundation for Statistical Computing.

[skag239-B22] Rousseeuw PJ. 1987. Silhouettes: a graphical aid to the interpretation and validation of cluster analysis. J Comput Appl Math. 20:53–65. 10.1016/0377-0427(87)90125-7

[skag239-B23] Rowan TN , Durbin HJ, Seabury CM, Schnabel RD, Decker JE. 2021. Powerful detection of polygenic selection and evidence of environmental adaptation in US beef cattle. PLoS Genet. 17:e1009652. 10.1371/journal.pgen.1009652 PMC8297814 34292938

[skag239-B24] Santana TEZ , Veroneze R, Menezes GRO, Rosa GJM. 2025. Comprehensive farm-level analysis of environmental and management descriptors for developing an efficient genetic evaluation of pasture-raised beef cattle. Transl Anim Sci. 9:txaf147. 10.1093/tas/txaf147 PMC12698201 41394299

[skag239-B25] Stewart WC et al 2020. Anthelmintic resistance in gastrointestinal nematodes and associated management factors in Intermountain West sheep flocks. Sheep Goat Res J. 35:30–37. https://digitalcommons.unl.edu/cgi/viewcontent.cgi? article=1022&context=icwdmsheepgoat (Accessed 3 December 2025).

[skag239-B26] Tremblay M et al 2016. Customized recommendations for production management clusters of North American automatic milking systems. J Dairy Sci. 99:5671–5680. 10.3168/jds.2015-10153 27157582

[skag239-B27] USDA-National Agricultural Statistics Service. 2025. Sheep and goats. U.S. Dep. Agric., Natl. Agric. Stat. Serv., Washington, DC. https://downloads.usda.library.cornell.edu/usda-esmis/files/000000018/zk51xc07n/9593wq66x/shep0125.pdf (Accessed 20 February 2025).

[skag239-B28] Venables WN , Ripley BD. 2002. Modern applied statistics with S. 4th ed. Springer.

[skag239-B29] Wickham H. 2016. ggplot2: Elegant graphics for data analysis. Springer-Verlag.

[skag239-B30] Yazar Gunes H et al 2026. Management practices across five major U.S. sheep breeds: producer survey insights. Transl. Anim. Sci. 10:txag041. 10.1093/tas/txag041 PMC13089442 42004578

