# Supplementary material for: Defining production environments in U.S. sheep populations: clustering climate conditions and management practices
Source: J Anim Sci. 2026 Aug 5;104:skag239. doi: 10.1093/jas/skag239 (PMC13668885; doi:10.1093/jas/skag239)
Supplement: Supplementary file 1 [file skag239_supplementary_data.zip › Supplementary Table S1.pdf]

Supplementary Table S1. Management survey questions showing significant differences ($P < 0.05$) across eco-management clusters by breed.

Survey section	Question ID	Katahdin	Polypay	Rambouillet-Targhee	Suffolk
General management	Q5	✓[1]			
	Q6	✓			
	Q8		✓		
	Q9	✓			
	Q10	✓			
	Q11			✓	
	Q12	✓			
	Q13	✓		✓	✓
Parasite management	Q14	✓	✓		✓
	Q15	✓	✓		
	Q16	✓	✓		
	Q17	✓	✓		✓
	Q18	✓	✓	✓	
	Q19		✓		
	Q20	✓	✓		
	Q23	✓			
	Q24	✓			
	Q25	✓	✓		
	Q26	✓	✓	✓	✓
	Q27	✓	✓		✓
	Q28	✓	✓		✓
Lambing management	Q31	✓			
	Q35		✓		
Udder health management	Q38	✓			
	Q40		✓		
	Q41		✓		
Culling management	Q44	✓			
	Q45	✓	✓		
Feeding management	Q46		✓		
	Q48		✓		
	Q49	✓	✓	✓	✓
	Q50	✓	✓		
Climate adaptation	Q54#1_3[2]	✓	✓		
	Q58	✓			

[1] A check mark (✓) indicates a statistically significant difference ($P < 0.05$) across eco-management clusters within breed.

[2] Q54#1_3 refers to Question 54 (housing structures used for most ewes during lambing; row c: "For lambing?"), response category 3.
