# Supplementary material for: Defining production environments in U.S. sheep populations: clustering climate conditions and management practices
Source: J Anim Sci. 2026 Aug 5;104:skag239. doi: 10.1093/jas/skag239 (PMC13668885; doi:10.1093/jas/skag239)
Supplement: Supplementary file 1 [file skag239_supplementary_data.zip › Sheep Producer Management Survey.pdf]

Sheep Producer Management Survey

Start of Block: Default Question Block

Q1 IRB Project ID #: 23225

Sheep Producer Management Survey

Dear Sheep Producers,

My name is Hilal Yazar Gunes. I am a Ph.D. student at the University of Nebraska-Lincoln (UNL) working with Dr. Ron Lewis. I am conducting a survey to better understand the impact of your farm or ranch environment on your animals' performance. Particularly, I am interested in how your management practices influence the robustness and climatic resilience of your flock, including traits like parasite resistance, lamb survival, udder health, and ewe longevity. To learn more about your practices, the survey that I prepared has the following sections: general management, parasite management, lambing management, udder health management, culling management, feeding management, and climate adaptation management.

If you are 19 years of age or older, engaged in the National Sheep Improvement Program (NSIP), and a producer of Katahdin, Polypay, Rambouillet, Suffolk or Targhee sheep, I invite you to participate in this research. Participation in this study will require approximately 30 minutes of your time to complete an online questionnaire through Qualtrics. There are no known risks to you from being in this research study. However, the information gained will help identify ways for improving robustness and climatic resilience in sheep flocks in the U.S. and enhance the value of the NSIP genetic evaluation to you and your customers.

We will not pay you to take part in this study. Reasonable steps will be taken to protect the privacy and the confidentiality of your study data. However, in some circumstances we cannot guarantee absolute privacy and/or confidentiality. The research records will be securely stored electronically through university approved methods and will only be seen by the research team and/or those authorized to view, access, or use the records during and after the study is complete. Those who will have access to your research records are the study personnel, the UNL Institutional Review Board (IRB), and any other person, agency, or sponsor as required by law, contract, or institutional responsibility. The information from this study may be published in scientific journals or presented at scientific and industry meetings and may be reported individually or summarized as a group. Your identity, however, will be kept strictly confidential. You may ask any questions concerning this research and have those questions answered before agreeing to participate in or during the study.

For study related questions, please contact the investigator(s):
Hilal Yazar Gunes: hyazargunes2@huskers.unl.edu
Dr Ron Lewis: rlewis5@unl.edu

For questions concerning your rights or complaints about the research contact the IRB at phone number 402-472-6965 or email address irb@unl.edu.

You can decide not to be in this research study, or you can stop being in this research study at any time before, during, or after the research begins for any reason. Deciding not to be in this research study or deciding to withdraw will not affect your relationship with the investigator, the UNL, or NSIP. You will not lose any benefits to which you are entitled.

You are voluntarily deciding whether to participate in this research study. By typing your name below, your consent to participate is implied. You should print a copy of this page for your records.

- ☐1 Are you over 19 (Yes or No) ____
- ☐2 Name ____
- ☐3 Today's date (mm/dd/yyyy) ____

---

*

Q2 Please provide your contact information in the given formats.

- ☐1Name ____
- ☐2 Address ____
- ☐3 Cell phone (xxx) xxx - xxxx ____
- ☐4 Landline if available (xxx) xxx - xxxx ____
- ☐5 Email (xxxx@xxx.xxx) ____
- ☐6 NSIP flock number (xxxxxx) _________________

Q3 May we contact you by phone for a follow-up interview?

- ☐1 Yes
- ☐2 No

Section A – General Management

Q4 Which of the following best describes the labor source in your flock? (Select one)

☐1 Self/family

☐2 Self/family and hired personnel

☐3 Hired personnel

Q5 In the past 12 months, how many ewes did you lamb in your operation?

☐1 15 ewes or less

☐2 16 to 30 ewes

☐3 31 to 50 ewes

☐4 51 to 100 ewes

☐5 101 to 250 ewes

☐6 More than 251ewes

Q6 In the past 12 months, how many ewe lambs did you retain or purchase as replacements?

☐1 15 ewe lambs or less

☐2 16 to 30 ewe lambs

☐3 31 to 50 ewe lambs

☐4 51 to 100 ewe lambs

☐5 101 to 250 ewe lambs

☐6 More than 251 ewe lambs

Q7 How many rams do you typically use for breeding?

☐1 2 rams or fewer

☐2 3 to 5 rams

☐3 5 to 10 rams

☐4 more than 10 rams

Q8 For how many breeding seasons do you typically use a ram?

☐1 1 breeding season

☐2 2 breeding seasons

☐3 3 or more breeding seasons

Q9 How many rams do you typically purchase in a year?

☐1 None

☐2 2 rams or fewer

☐3 3 to 5 rams

☐4 5 to 10 rams

☐5 more than 10 rams

Q10 How many times do your weigh your lambs through one year of age?

☐1 Once

☐2 Twice

☐3 Three times

☐4 More than three times

Q11 Do you first breed ewes as lambs or as yearlings (i.e., ewe between 1- and 2-years of age)?

☐1 Lambs

☐2 Yearlings

☐3 Depending, some as lambs while others not until yearlings

Q12 If you have a woolled breed, when do you shear your ewes (click all that apply)?

☐1 Spring (e.g., pre-lambing)

☐2 Late summer or fall (e.g., pre-breeding)

☐3 Do not shear

Q13 What percentage of ram lambs in your flock are castrated?

☐1 None (all kept intact)

☐2 Less than 25%

☐3 25 to 50%

☐4 More than 50%

Section B – Parasite Management

Q14 Do you deworm your flock (ewes and/or lambs)?

☐1 Yes
☐2 No

Display This Question:

Do you ever deworm your flock (ewes and/or lambs)? = *Yes*

Q15 How do you determine which animals to deworm?

☐1 FAMACHA scoring

☐2 Fecal egg counts

☐3 Observation of poor doers or other signs of disease (low productivity, bottle jaw, diarrhea, etc.)

☐4 No specific determination, and instead deworm all

☐5 Other (specify: __________)

Display This Question:

Do you ever deworm your flock (ewes and/or lambs)? = *Yes*

Q16 Describe your deworming regime:

☐1 Only use one dewormer

☐2 Use one dewormer until it no longer works, then switch

☐3 Alternate dewormers yearly

☐4 Alternate dewormers during the same season

☐5 Use a combination of dewormers each time

☐6 Other (specify: ________________)

Display This Question:

Do you ever deworm your flock (ewes and/or lambs)? = *Yes*

Q17 In the past 12 months, which dewormer(s) did you use? (Check all that apply)

☐1 Avermectins or Milbemycins (for example, Ivomec® Cydectin® Dectomax®)?
☐2 Benzimidazoles (for example, Panacur®/Safeguard®/Valbazen®)?
☐3 Imidazothiazoles (for example, Levasole®-levamisole)?
☐4 Benzenesulphonamides (for example, Curatrem®, Ivomec Plus®)?
☐5 Tetrahydropyrimidines (for example, Rumatel®)?
☐6 Other Method (Please state) ____

Display This Question:

Do you ever deworm your flock (ewes and/or lambs)? = *Yes*

Q18 Do you use any of the following products to treat for worms (do not include treatment for coccidia):

☐1 High tannin containing concentrate hay or pellets (for example, lespedeza, birdsfoot trefoil, sainfoin)

☐2 Diatomaceous earth

☐3 Botanicals/herbs/cayenne pepper/garlic

☐4 Copper oxide wire particles (COWP)

☐5 Feed a biological control product such as BioWorma® or Livamol® (*Duddingtonia flagrans*)

☐6 Other? (Specify: __________________)

Q19 Do you do any of the following as part of your internal parasite control program (check all that apply):

☐1 Change lambing season to reduce the risk of high parasite exposure

☐2 Provide supplement to increase resistance

☐3 Remove from pasture during high times of infection

Q20 Which of the following best describes the total amount of money you spent on deworming products (both pharmaceutical and alternative/natural) administered to sheep on your operation during the previous 12 months?

☐1 Less than $100

☐2 $100 to $300

☐3 $300 to $500

☐4 More than $500

Q21 Did you seek veterinary intervention for internal parasite-related issues in the past 12 months?

☐1 Yes, please specify the number of visits required _______________ and approximate cost per visit $_______________

☐2 No

Q22 Approximately how many days per year do you spend assessing and treating your flock for internal parasitism?

_______________ days per year assessing

_______________ days per year treating

Q23 Of lamb losses, approximately what percentage is due to internal parasites (through marketing or first breeding)?

☐1 Fewer than 5%

☐2 5 to 15%

☐3 16 to 30%

☐4 More than 30%

Q24 Through marketing or first breeding, what percentage of lambs do you think are poor doers because of internal parasites?

☐1 Fewer than 5%

☐2 5 to 15%

☐3 16 to 30%

☐4 More than 30%

Q25 When do you administer your first treatment for internal parasites to your lambs?

☐1 Never or rarely treated

☐2 Prior to weaning (e.g., within a month of birth)

☐3 Around weaning

☐4 When first place pasture

☐5 When pasture is rotated

☐6 When first deemed needed

☐7 When recommended by a veterinarian

Q26 How many times do you typically deworm lambs through marketing or first breeding?

☐1 Never or rarely

☐2 Once

☐3 Twice

☐4 Three times

☐5 More than three times

Q27 When do you deworm ewes during the annual production cycle? (Check all that apply)

☐1 Never or rarely

☐2 At lambing

☐3 At breeding

☐4 At weaning

☐5 When pasture is rotated

☐6 When deemed needed

☐7 When recommended by a veterinarian

☐8 Other (specify: ……………………………)

Q28 Are replacements from outside of the flock dewormed before being turned into the flock?

☐1 Yes, all replacements

☐2 Yes, a proportion of replacements

☐3 No

☐4 Not applicable since do not purchase outside replacements

Q29 Do you select for parasite resistance?

☐1 Yes

☐2 No

Display This Question:

Do you ever select for parasite resistance = *Yes*

Q30 What approach or tool would you use as a base for selection?

☐1 Phenotypic performance (FEC or FAMACHA scores)

☐2 Estimated Breeding Values (EBV) for FEC

☐3 A combination

☐4 Other (specify: …………………………)

Section C – Lambing Management

Q31 Which of the following best describes the area ewes give birth in your flock? (Check all that apply)

☐1 Individual lambing pens/jugs

☐2 Barn or shed (covered but without individual pens)?

☐3 Special lambing pasture that allows increased observation and/or shelter?

☐4 Other fenced pasture?

☐5 Open range?

☐6 Dry lot (pen which does not allow grazing)?

☐7 Other? (Specify: ________________________________)

Display This Question:

If answer Q31 with individual lambing pen/jugs

Q32 For how many days are ewes and their lambs kept in the jug?

---------------------- days in jug

Q33 Are the first-lambing ewes (e.g., ewe lambs, yearling ewes) managed separately from the mature ewe flock until after they lamb?

☐1 Yes

☐2 No

Q34 If you turn your ewes with lambs out to pasture, how soon after parturition?

☐1 Lambs do not go to pasture

☐2 Born on pasture

☐3 Shortly after birth

☐4 Before 30 days of age

☐5 Between 30 and 60 days of age

☐6 Between 60 and 90 days of age

☐7 Over 90 days of age

Q35 What percentage of newborn lambs receive colostrum by:

☐1 Hand feeding only: lambs were separated from the mothers immediately after birth and hand fed (i.e., Teat feeder/bottle/tube feeder)? __________ %

☐2 Both nursed the ewe and hand fed? __________ %

☐3 Nursed only _________ %

Q36 Do you hire extra labor during lambing season?

☐1 Yes

☐2 No

Q37 During the most recent lambing season, excluding nighttime, how frequently were lambing areas checked for newborns?

☐1 Continuous monitoring (including video)

☐2 Every hour

☐3 Every two hours

☐4 Every four hours

☐5 Twice daily

☐6 Once daily

☐7 Occasional monitoring (pasture lambing)

Section D – Udder Health Management

Q38 Do you select for improving udder health?

☐1 No

☐2 Yes (If so, specify how: _______________)

Q39 What percentage of your lambing ewes had clinical mastitis (abnormal milk or swollen udder) in the previous 12 months?

☐1 Less than 5%

☐2 5 to 10%

☐3 10 to 30%

☐4 Over 30%

Q40 What percentage of your lambing ewes were culled primarily due to mastitis (clinical or subclinical)?

☐1 Less than 5%

☐2 5 to 10%

☐3 10 to 30%

☐4 Over 30%

Q41 Which of the following best describes the total amount of money you spent on mastitis treatment products (both pharmaceutical and alternative/natural) administered to sheep on your operation during the previous 12 months?

☐1 Less than $100

☐2 $100 to $300

☐3 $300 to $500

☐4 More than $500

Q42 Did you seek veterinary intervention for udder health-related issues in the previous 12 months?

☐1 No

☐2 Yes, please specify the number of visits required _______________ and approximate cost per visit $_______________

Q43 Approximately how many hours did you spend treating udder health issues per ewe?

_______________ hours per ewe

Section E – Culling Management

Q44 Do you cull your ewes at a specific age? If so, at what age (in years)?

- ☐1 No
- ☐2 Yes, Specify: _______________

Q45 What are the three primary reasons you cull ewes in your flock? (Please rank from 1 to 3, with 1 the primary reason)

- ☐1 Age _______________
- ☐2 Teeth/Mouth problems _______________
- ☐3 Udder Health problems _______________
- ☐4 Reproductive problems (Failed to lamb or failed to wean lamb) _______________
- ☐5 Foot/Leg problems _______________
- ☐6 Respiratory problems _______________
- ☐7 Thin/unthrifty (Low body condition) _______________
- ☐8 Temperament/behavior _______________
- ☐9 Less desirable genetics (e.g., unfavorable EBV) _______________
- ☐10 Illness _______________
- ☐11 Economic Issues (for example, drought, market conditions etc.) _______________
- ☐12 Not listed above: Specify: _______________

Section F – Feeding Management

Q46 How do you feed your flock throughout the year?

☐1 Prepared diets

☐2 Pasture/rangeland only

☐3 Both

Display This Question:

How do you feed your flock throughout the year? = *Rangeland or Both*

Q47 How many months of year do you graze your flock on pasture or range?

☐1 1 month or less

☐2 1 to 3 months

☐3 3 to 6 months

☐4 More than 6 months

Display This Question:

How do you feed your flock throughout the year? = *Rangeland or Both*

Q48 What is your grazing resource (check all that apply)?

☐1 Irrigated pasture

☐2 Cropland

☐3 Native grassland

Q49 What is the total acreage grazed?

☐1 Less than 10 acres

☐2 10 to 50 acres

☐3 50 to 100 acres

☐4 100 to 500 acres

☐5 500 to 1000 acres

☐6 More than 1000 acres

Q50 Do you graze your sheep with any other livestock species? If so, what other species?

☐1 No

☐2 Yes, Specify: ………………

Q51 Do you provide mineral/vitamin supplements to your flock? If so, how are the supplements provided?

☐1 Yes, on the ground (floor) or on top of hay

☐2 Yes, mixed in the diet

☐3 Yes, in a self-feeder

☐4 No mineral/vitamin supplement provided

Q52 Do you provide protein/energy supplements to your flock? If so, how are the supplements provided?

☐1 Yes, on the ground (floor) or on top of hay

☐2 Yes, mixed in the diet

☐3 Yes, in a self-feeder

☐4 No protein/energy supplement provided

Q53 What source of water is available to your flock (check all that apply)?

☐1 Hand filled water troughs or buckets

☐2 Automatically filled water troughs

☐3 Stream or other natural water sources

Section G – Climate Adaptation Management

Q54 Which of the following housing structures were used for most ewes:
[Check one only for each row]

	Structure with four walls and roof	Structure with roof and three or fewer walls, for example, loafing shed (including transportable shade)	Forested area	Wind break (natural or planted)	No structure
a. During winter?	☐1	☐2	☐3	☐4	☐5
b. During summer?	☐1	☐2	☐3	☐4	☐5
c. For lambing?	☐1	☐2	☐3	☐4	☐5

Q55 Which of the following best describes the surface moisture of the ground or flooring that sheep stood on most of the time: *[Check one only for each row]*

	Usually dry	Wet about half the time	Almost always wet, but no standing water	Usually standing water or slurry
a. Summer/Fall?	☐1	☐2	☐3	☐4
b. Winter/Spring?	☐1	☐2	☐3	☐4

Q56 Do you provide additional supplemental feed when the weather is cold?

☐1 No

☐2 Yes

Q57 Have you recently changed or considered changing any management practices to cope with weather-related issues?

☐1 Yes, have already implemented

☐2 Yes, being considered

☐3 No

Q58 Do you seasonally transfer animals to another site?

☐1 No

☐2 Yes

Specify: ………………………. (Distance between sites in miles)

Specify: ………………………. (Zip code of the transferred site (to align with climate/weather information))

Display This Question:

If Do you seasonally transfer animals to another site? = Yes

Q59 How long are they staying at the new site?

☐1 Less than 2 months

☐2 2 to 4 months

☐3 4 to 6 months

☐5 More than 6 months

Display This Question:

If Do you seasonally transfer animals to another site? = Yes

Q60 How do you transfer them?

☐1 By truck

☐2 Herding on foot
